# Voters and the IMF: Experimental Evidence From European Crisis Countries

**DOI:** 10.1177/00104140231204229

**Published:** 2023-10-13

**Authors:** Evelyne Hübscher, Thomas Sattler, Markus Wagner

**Affiliations:** 1 47797 Central European University, Vienna, Austria; 2 27212 University of Geneva, Geneva, Switzerland; 3 27258 University of Vienna, Vienna, Austria

**Keywords:** international organizations, conditionality, fiscal austerity, sovereignty, credibility, public opinion

## Abstract

IMF interventions are often associated with rising political discontent in countries where the Fund intervenes. Studies examining this relationship, however, face the challenge of disentangling the impact of the IMF from the impact of the crisis that triggered the intervention. To address this challenge, we conduct survey experiments in Greece, Ireland, Portugal, and Spain and directly assess how voters evaluate the costs and benefits of an IMF intervention. We find that voters believe that the crisis will more likely be solved when the IMF intervenes, but they are also critical of the corresponding loss of national sovereignty. Because the former consideration, on average, dominates their assessment, IMF interventions increase the support of voters for unpopular economic policies. Nonetheless, cross-country differences suggest that continued public support for intervention hinges on the IMF’s ability to deliver on its promise to help resolve the crisis.

## Introduction

Political research has long debated to what extent globalization and democracy are compatible. Much of this research highlights the gains from international cooperation: joint efforts to tackle transnational problems provide more effective solutions. Since these joint solutions make the cooperating countries better off, citizens should support the delegation of power to international organizations. The success of anti-globalist political parties has, however, led to a reassessment of how voters evaluate the relative costs and benefits of international integration (e.g., Hooghe et al., 2019; Bearce & Scott, 2019; De Vries et al., 2021; Voeten, 2021). This research suggests that many voters perceive the sovereignty costs, that is, the reduced ability of countries to decide freely on key aspects of social and economic life, as more substantial than often assumed.

This tension between efficiency and sovereignty is particularly strong in times of economic crisis. In such situations, governments often ask for assistance from international organizations, especially the IMF. The involvement of an external actor enhances the credibility of economic reforms and allows for a swifter resolution of the crisis (Stone, 2002; Gray, 2013; Gehring & Lang, 2020; Alexiadou et al., 2022). But the outsourcing of decision-making to a non-majoritarian institution also raises concerns about the loss of control to a non-elected actor, which results in a “democratic deficit” (Berman, 2017; Bertsou and Caramani, 2020; Caramani, 2017; Ruiz-Rufino & Alonso, 2017). This potentially increases political resistance against reform policies, which may again undermine their credibility (Shim, 2022).

A crucial question in this debate is how voters perceive the trade-off between credibility and sovereignty when the IMF intervenes. Voters and their perceptions of external interference in domestic, macro-economic decision-making processes matter directly for assessments of democratic deficits and the related backlash against international organizations. If voters overwhelmingly reject such external interference, this increases dissatisfaction with the selected policies and points to a mismatch between voter attitudes and actual policy choices. This dissatisfaction, in turn, is at the core of the decision to vote for anti-globalist parties.

Our analysis, thus, has two main goals. First, it empirically estimates the causal effect of IMF presence on voters’ evaluations of the policy decisions. Since the literature yields contradictory predictions about the direction of this effect, the results will shed light on how voters reason about IMF interventions. Second, we empirically examine the relevance of the different possible mechanisms underlying voter evaluations. This allows us to better understand why the causal effect goes in one direction or the other. Our analysis focuses on the public support for fiscal adjustment because disagreement with these policies is at the core of political disruptions that the IMF may cause.^1^

So far, empirical studies of the political impact of the IMF tend to conclude that voters see IMF interventions negatively: the presence of the IMF leads to a decline in political support for incumbents, creates government instability, and reduces satisfaction with democracy (Alonso & Ruiz-Rufino, 2020; Bojar et al., 2022; Dreher & Gassebner, 2012). This suggests that voters perceive the costs of IMF interventions as more substantial than their benefits. Relatedly, research suggests that IMF interventions may occur strategically to shift the blame for unpopular policies toward an external actor (Vreeland, 1999; Smith and Vreeland, 2006). Although this may be politically beneficial for the government, it possibly further undermines support for the reform policy, satisfaction with democracy, and the legitimacy of the international organization (Biten et al., 2022).

A key challenge of existing empirical analyses is the difficulty of disentangling the impact of the IMF from the impact of the economic crisis that led to the intervention in the first place. When governments turn to the IMF, they typically face serious economic problems and have difficulty borrowing money on capital markets. Under these constraints, governments would have to adjust economic policy in any case, also without an intervention by the IMF. An analysis of the causal effect of IMF interventions on voters, therefore, requires that we identify how voters would have reacted to a similar policy in a similar situation without the IMF.^2^ Although the literature has made significant efforts to take this self-selection process into account (Przeworski and Vreeland, 2000; Stone, 2008; Copelovitch, 2010a; Chwieroth, 2015), the identification of the impact of the IMF on political and economic outcomes is difficult using observational data.

To address this challenge, we conduct survey experiments in Greece, Ireland, Portugal, and Spain that randomly expose respondents to different crisis resolution scenarios. This approach has two advantages. First, the experimental design allows us to estimate the causal effect of the IMF on voter evaluations and isolate this effect from other potential mechanisms that generally are associated with IMF interventions, for example, the severity of the crisis. Second, it also allows us to probe different causal mechanisms through which IMF interventions influence voters, in particular the credibility and sovereignty mechanisms highlighted above. With the help of a causal mediation analysis, we study how exactly voters judge the costs and benefits of IMF interventions and how different considerations countervail each other.

Contrary to the previous, observational studies, our key finding is that an IMF intervention has an overall *positive* effect on the level of support for an unpopular economic policy. On average, voters are more likely to support a fiscal adjustment package with rather than without IMF involvement. The main mechanism behind this result works through the IMF’s impact on economic credibility: voters expect that the crisis is more likely to be solved if the IMF intervenes, and this translates into greater support for the adjustment policy. At the same time, voters are aware of the constraints that the IMF imposes on national sovereignty. IMF interventions negatively influence citizens’ perceptions of the government’s room to maneuver, which translates into lower levels of support for fiscal adjustment. Taken together, however, the expectation that the crisis can be resolved with the IMF generally dominates the dissatisfaction over the loss of political control.

Our multi-country study also gives an indication of the scope conditions of our results. We find that voters in Ireland react most positively to an IMF intervention, while voters in Greece are very skeptical. This discrepancy exists because Irish voters have a strong expectation that the presence of the IMF will help to resolve the crisis, and this by far exceeds their worries about sovereignty. In contrast, Greek voters do not think that the IMF will help to end the crisis, and they associate the intervention more with a loss of democratic control. These differential assessments are consistent with the diverging experiences of these countries with external interventions during the Euro crisis. The impact of IMF interventions on policy approval, thus, ranges from no effect to a positive overall effect, and it is never negative. Given the very diverse prior experiences with the IMF from Greece to Ireland, we expect that the estimates for other European countries would also fall within this range.

## A Voter Perspective on IMF Interventions

International financial institutions such as the IMF play an important role for global economic policymaking. When countries face a sovereign debt crisis, the IMF can intervene and provide an emergency loan. Such an IMF program takes the form of a contract between the country and the IMF that sets out the requirements for getting the loan and the payback modalities. These modalities include a set of conditions that aim at addressing the problems that the IMF sees as the cause of the crisis. The conditions can vary, for example, by the degree of adjustment, the areas and timing of reform, or the payback details (e.g., Vreeland, 2007; Stone, 2008; Dreher, 2009; Copelovitch, 2010a; Dreher et al., 2015). Public spending cuts usually are among the most important conditions, but over the past decades the IMF has increasingly demanded broader adjustments, such as labor market flexibilization (Caraway et al., 2012). We will give more detailed examples of these programs below when we discuss IMF involvement in the countries that we examine empirically.

The profound impact that the IMF has on domestic policymaking puts it at the center of debates about the democratic legitimacy of supranational governance and the popular backlash against international organizations (e.g., Hooghe et al., 2019; De Vries et al., 2021; Voeten, 2021). Voters and their agreement or disagreement with the policies demanded by the IMF are crucial for democratic deficits and the popular discontent that arises from international integration. Our analysis contributes to this debate by examining how voters evaluate the costs and benefits of IMF interventions and, relatedly, to what extent public evaluations of economic policymaking are *input-* or *output-*oriented (Konstantinidis et al., 2022). An IMF program presents voters with a trade-off between faster economic stabilization, on the one hand, and a loss of control, on the other hand. The next two sections discuss these two countervailing mechanisms in detail.

### The IMF and Economic Stability

When governments turn toward the IMF, they usually face an economic crisis and find it difficult to borrow on financial markets. This is the case when investors doubt that the government will repay public debt and hence are hesitant to lend additional money. Examples of such crises include the Latin American debt crisis of the 1980s or the European debt crisis of the past decade.

A key challenge for governments in crisis countries is to convince investors that they have the political ability and willingness to restore financial stability. They can do so by implementing fiscal adjustments that increase public revenues and/or decrease public expenditures. But since these policies have serious, detrimental effects on politically important groups, investors doubt that the government is able to follow through with these measures (Barta, 2018; Hübscher & Sattler, 2017), undermining the credibility of announced policies (Drazen & Masson, 1994; Shim, 2022). Often, investors also lack more detailed information about the country and the government. They then judge economic credibility not based on what governments do but based on simple heuristics, such as their views of similar countries (Brooks et al., 2015) or ideology (Barta & Johnston, 2018; Sattler, 2013). These circumstances make it very difficult for governments to overcome the crisis on their own.

In such a situation, the IMF can help to enhance the economic credibility of the government (Fischer, 1999; Stone, 2002). International institutions can serve as a commitment device to lock in reform policies and as a scapegoat to reduce the political opposition against reforms (Baccini & Urpelainen, 2014; Smith & Vreeland, 2006; Vreeland, 1999). The IMF also provides a “seal of approval” when investors lack information about a country (Gray, 2013) and signals that the government is willing to incur a cost, the loss of autonomy, to solve the crisis (Alexiadou & Gunaydin, 2019; Alexiadou et al., 2022). The IMF then enhances credibility even if the content of a policy does not change. Relatedly, many voters in crisis countries are disappointed with their political elites. An external actor that is detached from domestic politics and who appears more competent than their own government can make these voters more optimistic about the resolution of the crisis.^3^ Voters, therefore, should become more confident that the crisis will be resolved when the IMF intervenes.

By this mechanism, voters should be more likely to support fiscal adjustments when the IMF intervenes. They have a strong interest in economic stability and often are willing to accept harsh measures in times of large economic uncertainty (Jurado et al., 2020). International constraints further strengthen governments in pushing domestic reforms through their discursive effect: they promote economic ideas that define what is possible and what the optimal economic decision is (Hay & Rosamond, 2002). This discursive power is particularly important in times of crisis when uncertainty is high. Citizens also are not per se opposed to independent experts (Bertsou, 2022). Many voters understand that the government needs to take steps to enhance its economic credibility, and the IMF can play an important role in this process. They should be more supportive of adjustment measures when the IMF intervenes because this strategy shows a way forward and out of the crisis.

This argument requires that voters see the IMF as a sufficiently neutral and competent actor. This may not always be the case because the IMF is not immune against political interference. For instance, IMF conditions tend to be softer and the loans bigger when banks from a large IMF shareholder country are exposed to a crisis, or when a crisis country plays an important geopolitical role, for example, in the UN Security Council (Copelovitch, 2010b; Vreeland, 2007; Dreher et al., 2015). These critiques raise concerns, but voters can still recognize the credibility gains even when they see the limitations of the IMF. What matters is the extent to which voters think that the political agenda undermines the IMF’s ability to increase confidence among investors. Especially voters who are disillusioned with the politics in their country should see the IMF’s isolation from domestic politics and its economic experience as an advantage.

The credibility argument takes an *output-*oriented perspective of political evaluations that highlights the effectiveness of a political decision. It suggests that the effect of the IMF on policy approval works through the economic credibility of a policy choice. In other words, the perceived impact of the IMF on credibility mediates policy approval because it explains a significant part of the variance in support for fiscal adjustment. Voters who do not believe that the IMF enhances credibility should not support fiscal adjustment more when the IMF intervenes. Voters who believe that the IMF increases credibility should also support fiscal adjustment more when the IMF intervenes.

H1a Voters are more optimistic that fiscal adjustment measures will effectively resolve the crisis when the IMF intervenes than when the IMF does not intervene.

H1b Voters are more likely to support fiscal adjustment when the IMF intervenes than when the IMF does not intervene.

### The IMF and National Sovereignty

The flip side of the credibility argument are the potential negative effects that IMF interventions have on voters’ perceptions of national sovereignty. As we now know, voters have become increasingly concerned about the impact of international integration on their countries (Mansfield et al., 2021; Walter, 2021). International institutions and their influence over domestic policymaking play an important role for these concerns.

International institutions have become more important because economic interdependence increases the need for international cooperation to address international problems. This is also the case for financial crises. In a globalized and interdependent world economy, a crisis in one country can threaten the economic stability of other countries and the international monetary system as a whole. To address this, the IMF organizes a coordinated response by providing the crisis country with additional financial resources. But in exchange, critical decisions are transferred to the international level where voters cannot influence them. This deprives voters of the idea that they participated in the decision process in a meaningful way (Berman, 2017; Bertsou and Caramani, 2020, 2022; Caramani, 2017). The delegation of power to an international institution, thus, stands in contrast to the idea of national sovereignty (Konstantinidis et al., 2019).

This is particularly salient for economic policies in times of crisis. IMF conditions always include policies that are politically contested. Fiscal adjustment programs, which are a central feature of most IMF programs, typically include spending cuts in major areas of the public budget, such as public pensions, unemployment benefits, public employment, or health care, and these cuts are generally unpopular among many voters (Hübscher et al., 2021, 2023a; Baccini & Sattler, Forthcoming; Bremer et al., 2020; Alonso & Ruiz-Rufino, 2020; Ciobanu, 2023). The IMF helps to override the political resistance against fiscal adjustment and tips the political balance in favor of pro-austerity voices. This is why the delegation of authority to a non-elected actor, such as the IMF, can be seen as a “challenge to democratic self-government” (Sánchez-Cuenca, 2020). Many voters may therefore perceive fiscal adjustment as a decision against the will of the people if it is implemented under an IMF program.

By this mechanism, voters should be less likely to support fiscal adjustment when the IMF intervenes. This does not mean that everybody objects to these policies. Many voters may regard austerity as the right choice (Barnes & Hicks, 2018). Fiscal adjustments are so contested because voter attitudes over these policies diverge rather than converge. When voters hold heterogeneous beliefs and have diverging interests, the sovereignty to decide over a policy in a competitive political process is particularly important. Besides the content of the selected policy, this process is valuable in itself because voters are more likely to take ownership of the adjustment measures when they had a meaningful choice (Konstantinidis & Reinsberg, 2020). In contrast, if this is not the case, voter satisfaction with policy outcomes and democracy more generally decreases (Armingeon & Guthmann, 2014; Ruiz-Rufino & Alonso, 2017).

The argument does not necessarily require that the policy adjustment is more severe when the IMF interferes. But the idea of sovereignty costs is also linked to the perception that the government may have taken a more balanced choice without the IMF because technocrats, like the IMF, “often come with a set of fixed ideas about the nature of the reforms to be adopted” (Alexiadou, 2020, p. 215). The IMF, for instance, has been characterized as a promoter of neoliberal ideas that uses its power to impose orthodox policies on the countries under an IMF program (Chwieroth, 2010; see also Hay & Rosamond, 2002 for a more general perspective). Other critiques see the IMF as a political actor that does not approach the crisis in an impartial manner but, for instance, represents specific creditor interests (Copelovitch, 2010b). Worries along these lines can raise further doubts about policies demanded by the IMF.

This argument again represents a two-step process that corresponds to an *input*-oriented perspective of policy evaluations: the IMF affects voter perceptions of sovereignty costs, which in turn mediate their assessment of fiscal adjustment. Voters who believe that the sovereignty costs of an IMF intervention are large should support fiscal adjustment less when the IMF intervenes. In contrast, voters who perceive sovereignty costs as small should not be less likely to support fiscal adjustment more when the IMF intervenes.

H2a Voters are more likely to perceive fiscal adjustments as going against popular will if mandated by the IMF.

H2b Voters are less likely to support fiscal adjustment when the IMF intervenes than when the IMF does not intervene.

### The Economic Stability—Sovereignty Trade-Off

Both mechanisms are theoretically plausible but point to countervailing effects that the IMF can have on voters. One argument is the flip side of the other because the two arguments are grounded in diverging assumptions about the reasoning of voters. Credibility requires that a voter sees the IMF as a sufficiently impartial, competent economic actor who effectively contributes to the resolution of a crisis. If this is the case, the voter may see the sovereignty costs as small and temporary compared to the long-term benefits. If, however, a voter has doubts about the IMF, she may be particularly worried about sovereignty costs because she expects that the country is better off if the policy response is decided by the government without the IMF.

This raises two questions that are crucial for our understanding of the impact of the IMF on voters. First, what is the overall effect of the IMF on policy approval? This question can be answered by testing the two competing hypotheses H1b and H2b against each other. If we find that the IMF decreases public support for fiscal adjustment, then this would be evidence that an IMF presence increases the democratic deficit and provides a motivation to vote for parties that want to roll back international integration. If, in contrast, the IMF increases public support for fiscal adjustment, this leads more optimistic conclusions about the role of the IMF.

Second, do voters reason about credibility and sovereignty as the two arguments propose? This question can be answered by testing hypotheses H1a and H2a. In addition, we can examine to what extent the impact of the IMF on each mediator variable, credibility and sovereignty, translates into more or less overall support for fiscal adjustment. For instance, it is possible that the effect of the IMF through both channels is equally large. In this case, the two countervailing effects on policy approval cancel each other out. It is also possible that the effect of the IMF on policy approval through one of the two channels is larger. If so, the analysis of the two mechanisms helps us to understand why the overall effect goes in one direction or the other.

Finally, the impact of the IMF on voters can also vary across contexts. The economic and social impact of IMF programs on countries varies considerably (Kentikelenis et al., 2016; Lang, 2021; Vreeland, 2003). How voters reason about the IMF possibly depends on the nature of politics in a crisis country, the origins of the crisis, or the past experience of voters with the IMF. The goal of the next sections, therefore, is to empirically examine the causal effect of the IMF on public approval of fiscal adjustments and to explore the empirical relevance of the different theoretical mechanisms. We will also explore the scope conditions of our findings to the extent that this is possible. The analysis covers very different countries that allow us to explore to what extent the importance of each mechanism differs across contexts.

## Research Design

### Empirical Strategy

Research on the political effects of IMF involvement faces an important challenge. To identify the causal effect of the IMF on voter attitudes and behavior, we need to compare a fiscal adjustment policy with the IMF to an identical (or similar) situation of fiscal adjustment without IMF involvement. For instance, if we compare an IMF intervention in a crisis situation to no IMF intervention in normal times, then we cannot disentangle the effect of the intervention from the effect of the crisis. Or, if we compare an IMF intervention that entails a fiscal adjustment package to a situation without the IMF and no fiscal adjustment, then we cannot disentangle the effect of the IMF from the effect of policy. This is problematic because voters may also punish governments for economic crises and fiscal spending cuts in situations when the IMF does not intervene.

Researchers who study the IMF are well aware of this problem. Most analyses today make a serious effort to address the problem that countries under an IMF program are different from countries without a program (Przeworski and Vreeland, 2000; Stone, 2008; Copelovitch, 2010b; Chwieroth, 2015). This research employs sophisticated empirical models that distinguish between different stages of the selection process into an IMF program. For instance, the models account for the government’s decision to demand financial support and the IMF’s decision to respond to the country’s request. Modelling this process helps to reduce the bias that arises from non-random selection into IMF programs, but it can only imperfectly address the resulting problems for causal inference.

We address this problem through survey experiments, which have two advantages. First, they allow us to present different scenarios to voters that are identical except for whether there is an IMF intervention or not. We do this by using vignette experiments embedded in population surveys. By randomly assigning respondents to either a scenario with the IMF or a scenario without IMF involvement, we can identify the causal effect of IMF interventions on the responses of voters. Second, survey experiments allow us to examine the political effects of IMF interventions at the individual level rather than relying on aggregate election results. In this way, we examine a crucial link in the mechanism that connects IMF interventions to public discontent in a country.

Our experimental design follows previous analyses of public opinion in international politics (Mattes & Weeks, 2019; Tomz & Weeks, 2013). We provided all respondents with a general scenario that takes place in the future, in 2026. Respondents were told that their country is experiencing an increase in the level of public debt and that the government is finding it more difficult to borrow money on financial markets. The head of government therefore announces that spending cuts will be implemented, which will affect funding for a broad range of areas, including public pensions, health care, education, and public transport. And finally, the main opposition party criticizes the measures and doubts whether they will be successful.

Within this general description, we randomly manipulated four aspects of the scenario, which yields a 2 × 2 × 2 × 2 experimental design. First and foremost, one group of respondents learned that the spending cuts are a condition of the IMF. Specifically, respondents in this group read: “The Prime Minister says that these spending cuts are necessary. This is because the International Monetary Fund (IMF) has made these cuts a precondition for [country] to get an emergency loan that could stabilize the financial situation. The IMF is an international organization that provides emergency loans to countries in crisis, but only to governments who commit to carry out certain reforms. [Country] would not receive the IMF loan without the cuts.” The statement about IMF involvement was omitted in the scenario presented to respondents of the other group.

We deliberately refer to the IMF instead of the EU, a specific EU institution (the EU Commission or the ECB) or the Troika, even though the EU played an important role for past financial interventions in the countries that we examine.^4^ Financial assistance is one of the IMF’s primary functions, and the IMF regularly intervenes in this manner in its member states. In contrast, emergency lending was an exceptional role that the EU took for the first time during the Eurozone crisis. Given the EU’s importance for many other issue areas, respondents have many other connotations when they think about the EU, which in turn affect the respondent’s general support or opposition toward EU policies. The IMF therefore better represents the type of organization that is central for our analysis of external interventions during financial crises.

In addition to the IMF intervention, we also manipulated the size of the debt increase and the seriousness of the economic situation; the size of the spending cuts and how seriously they affect public spending; and the partisanship of the government and the main opposition party. The latter was varied between the two largest parties—at the time—in the countries included in our survey.^5^ We do this because respondents may associate IMF interventions with more serious crises, greater spending cuts or a particular type of government. By manipulating these dimensions, we can isolate the impact of the IMF from these other factors that often covary with the IMF. At the end of the experimental part, we summarized the situation again for the respondent using four bullet points, one for each treatment. Table 1 summarizes these four treatments. The exact wording and setup of our experiment can be found in the online supplementary material (Appendix A).

**Table 1.** Treatments.

Treatments	Attributes of treatment
IMF intervention	[yes] [–]
Government partisanship	[right] [left]
Size of proposed cuts	[moderate] [large]
Severity of crisis	[moderate] [large]

After the description of the scenario, we asked respondents to evaluate the government’s decision to adjust public spending. Specifically, respondents were asked: “To what extent would you approve of the Prime Minister’s announcement to impose spending cuts in response to the debt crisis?” They could then respond using a slider on an 11-point scale where 0 means “strongly disapprove” and 10 means “strongly approve.” This question is used as our outcome variable to test H1b and H2b.^6^

To test hypotheses H1a and H2a, we ask two additional outcome questions. To test the credibility mechanism, we asked whether respondents “think that the government’s decision to cut spending will be successful or unsuccessful in resolving the debt crisis?”. For the sovereignty mechanism, we asked respondents to what extent they “think that the government was free to choose its own response to this debt crisis?”. Respondents indicate their position on 11-point scale (0–10), with higher values representing greater credibility and sovereignty, respectively.^7^

Finally, we conduct a causal mediation analysis along the lines proposed by Imai, Keele, and Tingley (2010) and Imai, Keele, and Yamamoto (2010) (for a similar approach, see Mattes & Weeks, 2019). This approach allows us to calculate the average causal mediation effect of each mechanism: it estimates the proportion that each mediator variable contributes to the overall variance in our main outcome variable, approval of fiscal adjustment. In other words, it examines if the impact of the IMF on policy approval works through credibility and/or sovereignty and hence links hypotheses H1a and H2a to hypotheses H1b and H2b.^8^

### IMF Interventions and Country Context

The survey experiment was conducted in the four countries that were at the center of the European debt crisis: Greece, Ireland, Portugal, and Spain. The survey was carried out in August 2020 and administered by Ipsos, which either used its own country-specific panels or collaborated with other firms to provide a large enough pool to sample respondents. Respondents were selected from these access panels using gender- and age-based quotas. The individual country-specific samples are restricted to voting-age nationals under the age of 70. In each country, we surveyed approximately 1,200 respondents.

We chose these four countries because the IMF intervention that we describe represents a plausible scenario for respondents in these countries. The IMF was an instrumental actor in all four countries during the European debt crisis and has been part of the public debate throughout the crisis. This gives respondents an idea what such an intervention entails for them and their country. This prior exposure, therefore, enhances the external validity of our analysis. In contrast, it would be unclear what an IMF program means for voters in countries that have never seen such a program and are unlikely to see one in the near future, for example, in Germany or the United States.^9^ This requires, however, that we interpret our survey results in light of these experiences and the varying, country-specific contexts. Table 2 provides basic information on the size of the rescue package and the cumulative austerity measures that were implemented as well as an overview of two key economic indicators, the average growth rate (2010–2015) and the average fiscal deficit (2010–2015) during the aftermath of the crisis and the level of economic growth and deficit in 2019, before the COVID-19 crisis.

**Table 2.** Bailouts, Austerity, Deficit, and Growth.

	Rescue package*	Austerity**	Deficit (*ϕ*)***	Deficit (2019)	Growth (*ϕ*)***	Growth (2019)
Greece	273.3	23.6%	−9.0%	.8%	−4.2%	1.9%
Portugal	78.0	16.8%	−7.0%	.1%	−.4%	2.7%
Spain	41.3	11.3%	−8.3%	−3.1%	0%	2.0%
Ireland	67.5	14.5%	−11.0%	.5%	6.1%	5.4%

Notes: * in billions (€); ** cumulated spending cuts and tax increases as % of GDP (2008-2015); *** average between 2010 and 2015. Sources: Devries et al. (2011); Alesina et al. (2019), IMF macro-economic indicators, and own analysis of IMF Reports.

The origins of the debt crisis differed significantly between Ireland and Spain on the one hand, and Portugal and Greece on the other. In Ireland and Spain, the crisis followed a construction and credit boom that led to a housing bubble, which—in the wake of the global financial crisis—resulted in a collapse of the domestic banking infrastructure. In a first response to this crisis, both countries designed packages to rescue banks and nationalized key mortgage lenders. Both economies experienced a significant increase in unemployment and emigration and a sharp rise in public debt. As a result, they called on the IMF/Troika for support. While the IMF intervention in Ireland took place in 2010, Spain first tried to address the problems on its own but had to resort to the IMF in 2012 under the pressure of international financial markets. The financial support for Spain came exclusively from the EU, but the deal and reform conditions were negotiated in close cooperation with the IMF. Both countries exited the program within a relatively short time frame (Ireland at the end of 2013, Spain at the beginning of 2014). Ireland’s economic recovery was smoother than Spain’s, which can be partially attributed to the high interconnectedness and financialization of the Irish economy. In Spain, different types of reforms helped to increase the share of exports, but youth unemployment and sluggish domestic consumption remained a problem.

Portugal and Greece struggled with structural economic problems already before the financial crisis. While the international financial crisis worsened the Greek situation, other predominantly domestic factors, such as large fiscal deficits, tax evasion, and tax avoidance, deepened the challenges the country faced. Greece turned to the IMF in 2010 for the first time after new data revealed the real extent of Greece’s public deficit and debt, which de facto excluded the country from private capital markets. The country turned to the IMF again in 2012 and 2015 and exited these programs only in 2018. Under the auspice of the IMF, Greece had to implement massive spending cuts that proved to be very challenging to sustain politically (Dellepiane-Avellaneda & Hardiman, 2015). In the subsequent years, the IMF programs were repeatedly re-negotiated and adjusted. The IMF also critically reflected on its interventions in a number of reports and conceded that the programs were partially detrimental and slowed down economic recovery (IMF, 2013). This is also indicated in Table 2 where Greece stands out as the clear laggard. In comparison, the depth of the crisis in Portugal was less severe, but the country also faced sharp pressure from financial markets and was unable to refinance government debt without the help of third parties. The Portuguese government turned to the IMF in 2011 and was part of a program until mid-2014.

Our multi-country approach has the advantage that we can explore the scope conditions of our findings. We will examine how voters in different countries perceive the costs and benefits of IMF interventions, at least in Europe, and hence what the possible range of effects of the IMF on voters is. We interpret these results in light of the different contexts at the end of the Results section.

**Figure 1.**
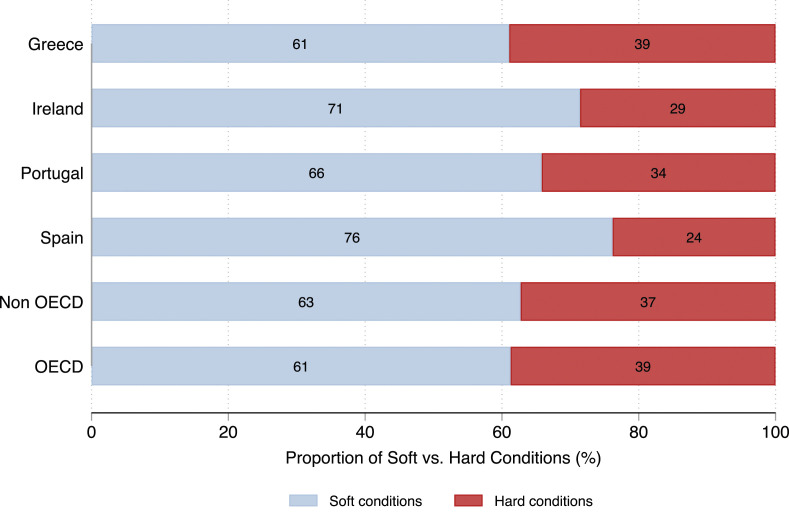
Proportion of hard versus soft conditions demanded by the IMF; Source: Kentikelenis et al. (2016) and own collection of data (for Spain).

**Figure 2.**
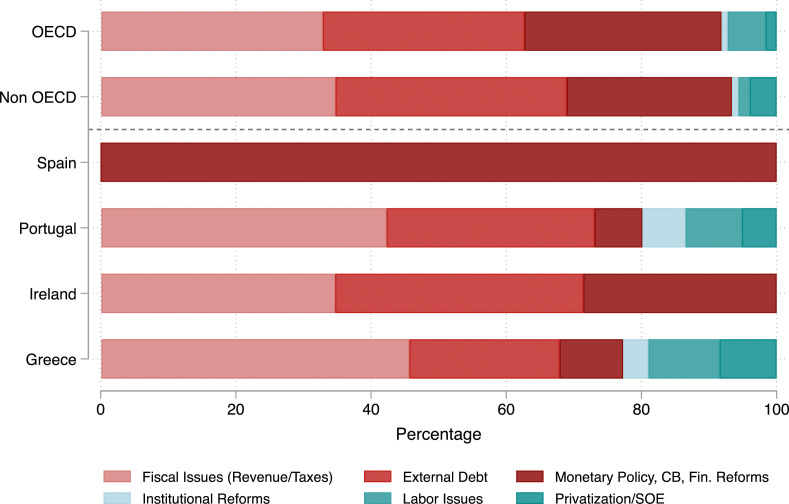
Reform conditions by policy dimensions (in %); Source: Kentikelenis et al. (2016) and own analyses.

**Figure 3.**
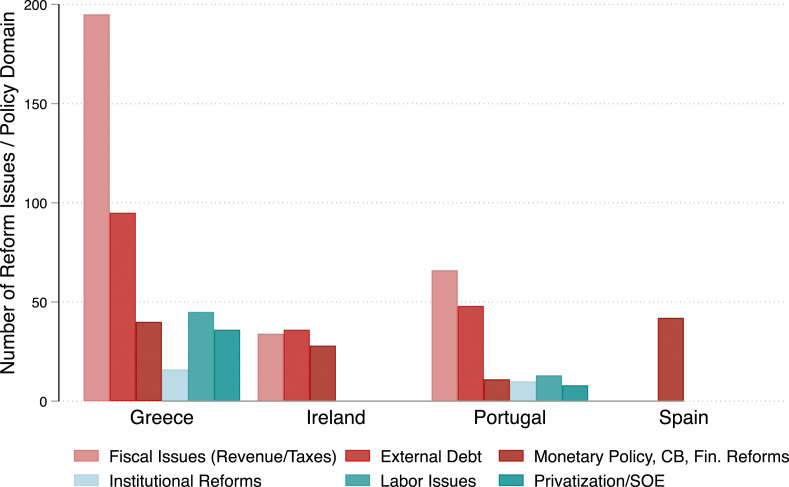
Reform conditions by policy dimensions (in absolute numbers); Source: Kentikelenis et al. (2016) and own collection of data (for Spain).

Another important aspect of the scope conditions is the nature of the IMF programs and their comparability to the programs in other countries. Figures 1–3, therefore, examine the design of IMF programs in our countries and those in other OECD and non-OECD countries between 2008 and 2014.^10^ As a first cut, we assess the distribution of hard versus soft conditions imposed by the IMF.^11^ Figure 1 shows that the share of soft versus hard conditions across the countries covered by our analysis and the average share of soft versus hard conditions in OECD and non-OECD. The share of hard conditions in the countries most hit by the Eurozone crisis (Greece and Portugal) closely resembles the pattern in the wider universe of cases (represented by other OECD countries and non-OECD countries). Ireland and Spain have slightly lower shares of hard conditions imposed by the IMF than the other countries.

Figures 2 and 3 show which policy areas were most affected by conditionality, first as the share of conditions in one area relative to other areas and second as the absolute number of conditions in a policy area. We differentiate between six core policy fields, three of them are closely related to the broader financial and fiscal architecture of a country (i.e., fiscal policy measures related to revenues and taxes, debt management, and financial regulations). The other three dimensions are linked to the institutional and regulatory fabric of a country’s economy (institutional reforms, labor market issues, and state interferences in markets). The majority of IMF conditions concerned a) fiscal policy issues (revenue and taxes) and b) the size and management of external debt. The third largest policy dimension was related to monetary policy, the regulation of the financial sector, and central banking. This confirms that the focus on fiscal adjustment in our survey experiment touches upon a highly salient component of IMF programs. The exception to this pattern is Spain, which was only subject to conditionalities related to its monetary policy.

In terms of country variation, we see again that Greece had to implement the highest numbers of reforms, of which almost half of the reform requirements were linked to fiscal issues. In order to reduce the fiscal deficit, the Greek government had to implement significant spending cuts, but also increase taxes. Portugal also faced a significant share of reforms related to fiscal policymaking, but the absolute number of conditions imposed in this sector was smaller. This is also the case for Ireland and especially Spain, whose bailout package was mostly designed to stabilize the Spanish banking system. Portugal and Greece also had to liberalize labor markets, privatize state-owned firms, and other specific services, such as telecommunications and energy, and implement other institutional reforms. Again, the number of conditions in Greece in these policy areas was larger than in Portugal.^12^

In sum, the composition of IMF programs in our four countries does not differ dramatically from programs designed for other OECD or non-OECD countries. Greece and Portugal had to implement more policies related to regulatory reforms than Ireland and Spain or other OECD and non-OECD countries undergoing IMF interventions during the same period. Overall, however, the comparison shows that our countries do not differ greatly from the universe of cases. We believe that this increases the confidence in our results and that they are generalizable, at least with respect to the nature of IMF conditions.

## Results

### Overall Effect

**Figure 4.**
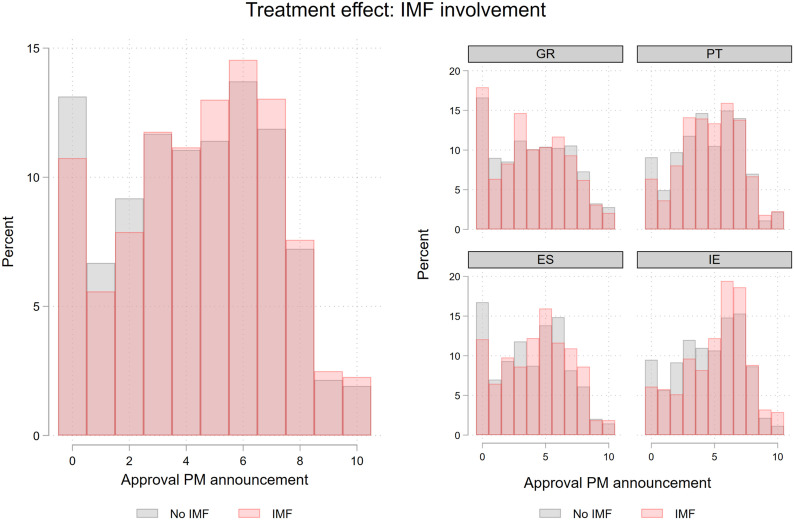
Distribution of IMF conditionality across different levels of voter approval for austerity package.

The main result of our experiment concerns the treatment effect of including IMF loan conditionality in the vignette, which corresponds to a test of hypothesis H1b against H2b. Figure 4 shows the difference in approval of fiscal adjustment for respondents who were exposed to a scenario in which adjustment is mandated by the IMF and respondents that were exposed to a scenario in which adjustment is announced without the involvement of the IMF.

The figure offers two key takeaways. First, the level of approval of fiscal adjustment, on average, is higher for respondents who were exposed to the IMF treatment than for respondents who were exposed to a vignette that does not mention the involvement of the IMF. This is the case for most increments of our dependent variable, but particularly pronounced for higher levels of approval. Second, when disaggregating the data and assessing approval of austerity for each country individually, we see a more nuanced picture. What stands out are the particularly high levels of approval of austerity in Ireland, especially in the case in which austerity is mandated by the IMF. A similar picture is present for Spain, whereas in Greece and in Portugal, the overall level of approval of austerity is lower on average, and the differences between the two groups are not as pronounced as for the other two countries.^13^

**Figure 5.**
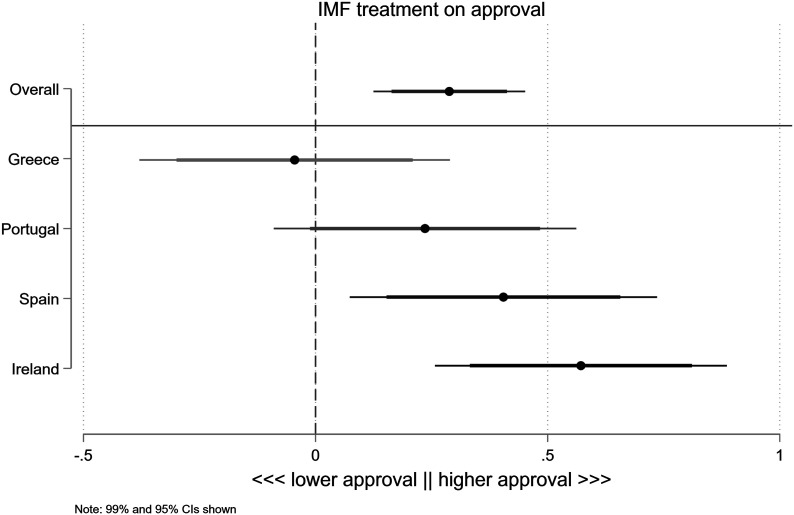
Effect of IMF conditionality on voter approval for austerity package.

In line with these results, Figure 5 shows that the IMF treatment has a statistically significant, positive effect on support for the adjustment package. Specifically, approval for that package increases by .3 units on the 0–10 scale when the IMF is involved.^14^ This effect is statistically significant at the .001 level for all four countries combined. This is equivalent to about ten percent of the standard deviation of the outcome variable (2.6 units). We also note that this effect is large compared to the other three treatments included in the vignette (see Appendix, section F.2). The “size of the debt” has no overall effect (*p* = .2), while the effect of the “size of the spending cuts” is about half the size of that of IMF involvement and statistically less significant (*p* = .1). The overall effect of the “party in government” is also not statistically significant (*p* = .68), though additional analyses show that, unsurprisingly, reactions to this treatment are strongly conditional on whether the voter is a supporter of the party in government.

While the overall treatment effect of IMF involvement is positive, it varies by country, with the clearest positive effect in Ireland and Spain. In Portugal, the response is also positive and similar in magnitude, if not statistically significant at the .05 level. Overall, the effects in Portugal, Spain, and Ireland are broadly similar. However, in Greece there is no positive, statistically significant response to the IMF.

In addition to these average effects, we examine how voters vary in their assessment of IMF interventions. As we discussed in the theoretical section, the two mechanisms rely on important assumptions about voters’ prior beliefs about the IMF. The heterogeneous treatment effects (HTEs) allow us to probe the importance of these assumptions.

**Figure 6.**
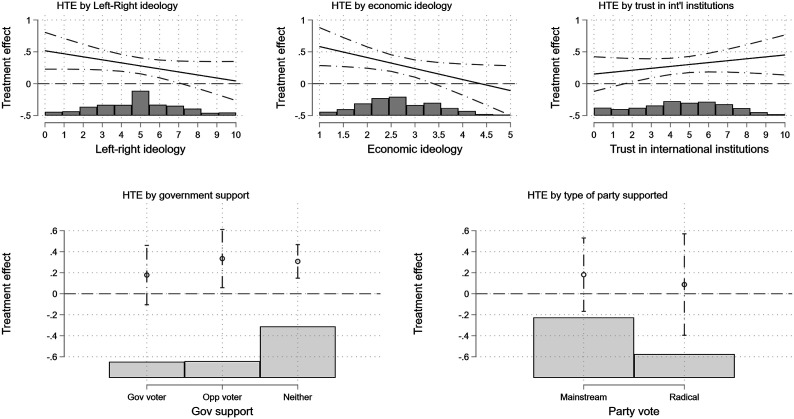
Treatment effects, by voter-level characteristics.

Figure 6 shows how five theoretically relevant voter-level characteristics moderate the treatment effect. Specifically, we focus on how general left–right and economic ideology, trust in international institutions, government or opposition support, and mainstream or radical party vote moderate the average level of approval of IMF conditionality. Left–right ideology is measured using the standard 0–10 left–right question (with 0 standing for the most leftist position, and 10 for the most rightist position one can take); economic ideology is measured using the mean response to five questions referring to different economic issues;^15^ trust in international institutions is measured using mean trust, on a 0–10 scale, toward the United Nations and the World Bank; and mainstream and radical party vote is measured using past vote choice.^16^ The models that examine the HTE for ideology control for the HTE for trust in international institutions (and vice versa), and the models that examine the HTE for radical vote control for whether one’s preferred party is in government in the experimental vignette.

Both left–right and economic ideology moderate the treatment effect in similar ways: on average, left-wing individuals are more likely to support the measures if the IMF is involved as opposed to a situation without IMF involvement. Note that this does not mean that left-wing voters are more supportive of adjustment packages. The effect only shows that—on average—more leftist respondents react more positively to our treatment than more rightist respondents. In fact, the average approval of austerity only increases for left-wing voters, whereas the treatment does not have an effect on the average level of approval for more conservative citizens. This indicates that the gap in the approval of austerity between left- and right-wing voters decreases in the presence of the IMF with leftist voters becoming more approving of austerity. A possible explanation of why more leftist voters are more supportive of the IMF could be that the need to involve the IMF sends a signal that austerity really is needed.^17^ Unsurprisingly, we can see that those who trust international institutions are more likely to react positively to IMF involvement. Note that only the interactions with left–right and economic ideology are statistically significant.

Figure 6 also presents heterogeneous treatment results by partisan support. While the differences in these HTEs are not themselves statistically significant at conventional levels, their direction does exhibit relevant patterns. The bottom left panel shows that those who do not support the governing party (which varied randomly in the vignette) are more likely to respond positively to IMF involvement. This is consistent with the notion that IMF involvement helps to garner support from individuals who would otherwise oppose government policies or do help to get the support from voters who do not trust the government but are confident that IMF conditionality can function as a commitment device. This may particularly apply to people who are supportive of the opposition. The bottom right panel shows, consistent with the HTEs for ideology, that radical party supporters are less likely to react positively to IMF involvement. Whereas supporters of the radical right may be more likely to oppose IMF involvement on sovereignty grounds, supporters of radical left parties may oppose IMF involvement on effectiveness grounds. We examine these mechanisms in more detail in the next section.

### Mechanisms

**Figure 7.**
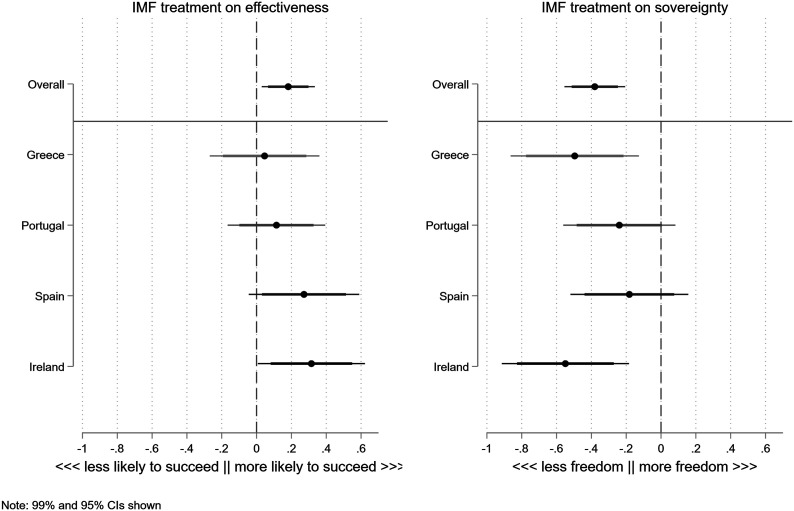
Treatment effects on mediators, by country.

We now turn the question of the mechanisms as represented by hypotheses H1a and H2a. We do this by examining the impact of the IMF treatment on the two additional outcome questions that capture the credibility and the sovereignty mechanism. Figure 7 shows that the IMF treatment has the expected effect on perceived effectiveness and perceived sovereignty. The overall model shows that IMF involvement increases the perceived probability that the crisis will be resolved by about .2 units. At the same time, IMF involvement reduces the perceived sovereignty of the national government: perceptions that the government was free to choose its own policy are about .4 units lower if the IMF treatment is mentioned in the vignette. We also find that perceived “effectiveness” and “sovereignty” themselves affect approval of fiscal adjustment (see Appendix Section F.3).^18^

**Figure 8.**
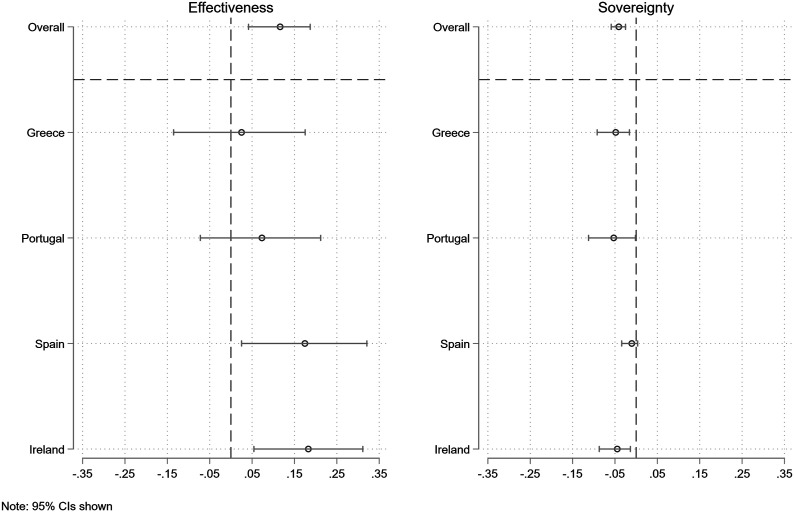
ACME for each mechanism, by country.

Next, Figure 8 presents the results of our causal mediation analysis. It shows that there is one dominant mechanism: voters approve of the policy because they believe that a package demanded by the IMF will be more effective in resolving the debt crisis. The average causal mediation effect of effectiveness is about .11, with 40% of the total effect mediated by this mechanism.

Interestingly, this positive mediating effect is counteracted slightly by the negative mediating effect of the IMF treatment on the perception that the national government was free to choose its own path (sovereignty). The average causal mediation effect of sovereignty is about −.04, with 14% of the total effect mediated by this mechanism. Hence, this statistically significant and negative mediation effect implies that IMF involvement makes respondents see the government as less sovereign, which leads to a reduction in the support for the policy package. However, this effect is not strong enough to balance the positive effect of IMF involvement on the perceived effectiveness of the program.^19^

Finally, Figure 8 also shows that these mediating effects are similar across contexts. Concerning perceived effectiveness, the main exception is Greece, where it is not via this mechanism that IMF involvement has an effect on approval of the policy package. Turning to the government’s national sovereignty, the effects are remarkably similar, even if the mediating effect for Spain is smaller than for the other countries.

In sum, we find that the key mechanism through which IMF involvement affects public attitudes toward fiscal adjustment is via perceived effectiveness. When voters learn that the policy package is part of an IMF program, they believe that this package will be more likely to resolve the crisis. At the same time, IMF involvement also reduces perceptions of national sovereignty, which in turn lowers approval for the policy package. However, this negative effect is not large enough to balance the positive impact of IMF involvement overall.

### Scope Conditions

Although the cross-country differences are not the central focus of our analysis, they are still useful to get a sense of the scope conditions of our findings. As we describe above, the composition of the IMF packages in the countries that we examine roughly correspond to IMF packages in other OECD and non-OECD countries. But the macro-economic context, roots of the crisis, and the path to recovery from the European debt crisis differed considerably.

The prior experiences of voters offer an explanation for the cross-country differences that we find. The assessment of the IMF is most positive in Ireland and Spain, the two countries that saw fewer conditions and conditions that were mostly tackling monetary and fiscal issues. The economies of these two countries also recovered more quickly. Citizens react more critically toward the IMF in Portugal and especially Greece. These two countries were subject to a higher share of hard conditions and also recovered more slowly. It is plausible that voters in our survey evaluate potential IMF programs in light of their memory of the past crisis and the role of the IMF during this crisis. This means that our respondents did not enter our experiment as entirely neutral subjects; instead, their differing “pre-treatment” experiences influenced their reactions. Considering that the IMF often intervenes in countries that already had IMF programs at an earlier stage, such prior experiences are an important feature of public reactions to the IMF.

Our multi-country approach, therefore, is useful to delimit the boundaries of the impact of the IMF on the public assessments of fiscal adjustments. We believe that the varying contexts from Greece to Ireland give an indication of the range of effects that are plausible, at least in Europe. The estimated effect ranges from no effect in Greece to a strong, positive effect in Ireland. It is never negative despite the widespread view that the IMF increases public discontent. Our results also show that credibility is an important aspect of IMF interventions, and voters in different contexts recognize this. In none of the countries do sovereignty concerns trump concerns about credibility.

## Conclusion

This paper examines how voters judge the credibility–sovereignty trade-off that characterizes the delegation of economic policymaking to non-elected, external actors, such as the IMF. Voter evaluations of this trade-off and their resulting approval, or disapproval, of hard economic choices are at the core of arguments about democratic deficits caused by the IMF and the backlash against international organizations.

Our results show that an IMF intervention in many instances increases approval of fiscal adjustment. This means that the effects of the IMF on voter evaluations is often more positive than is generally assumed. Our results differ from previous research (e.g., Alonso & Ruiz-Rufino, 2020; Bojar et al., 2022; Dreher & Gassebner, 2012) because our experimental approach allows us to disentangle the impact of the IMF from the impact of the economic situation of the country that needs a rescue package. Although voters are dissatisfied with the loss of democratic control, their expectation that the IMF helps to resolve the crisis dominates in different European countries.

The multi-country analysis also gives an indication of the scope conditions of our results. The positive effect of the IMF on policy approval is strongest in Ireland, weaker in Spain and Portugal, and non-existent in Greece. It is never negative. Given the experience of Greece with externally imposed policies during the European Debt Crisis, we interpret the results from Greece as a “lower bound” of the effect in European countries. To what extent these results are generalizable to non-European and non-OECD countries is something to be explored in future research. Often, the situation in these countries more resembles the situation in Greece than the one in Ireland. This potentially means that the IMF is viewed less positively in these countries. Future research could also examine which factors, such as the democratic tradition, length of EU membership, and other institutional or economic characteristics, moderate the IMF’s effect on voter approval. Such an analysis requires a different research design because these factors primarily vary on the country level. With only four countries, our research design is better suited to zooming in on mechanisms and not on the conditioning factors.

Our analysis identifies the impact of IMF presence holding other factors constant. For instance, the experiment allows us to estimate the effect of the IMF that is unrelated to the effect of greater fiscal adjustment on public evaluations. Respondents, however, may still associate the IMF with a different composition of the adjustment package in our analysis, something that we do not control for. We also note that policy approval drops in our analysis if the size of adjustment increases. The IMF may also have other negative effects, for example, on social inequality (Lang, 2021). To the extent that the IMF demands harder adjustment measures than would have occurred otherwise, the negative effect of additional adjustment on political support can reduce the positive effect of IMF presence through economic credibility.

Our analysis also points to the limits of “technocratization” of economic policy. Our results suggest that voters in fact are conscious of the democratic challenges that come with technocratic solutions. Voters may not tolerate losing political control over core issues of macro-economic decision-making in normal times. Relatedly, an important question concerns the downstream electoral effects of policy approval. We found that IMF involvement, or international integration more generally, reduces perceived national sovereignty, which in turn reduces economic voting (Hellwig & Samuels, 2007; Costa and Pannico, 2021). The increased support for the policy package may therefore not translate into electoral benefits. Indeed, we find weaker effects for vote choice in our analyses (as indicated in section F of the online appendix).

## Supplemental Material

Supplemental Material - Voters and the IMF: Experimental Evidence From European Crisis Countries Supplemental Material for Voters and the IMF: Experimental Evidence From European Crisis Countries by Evelyne Hübscher, Thomas Sattler, and Markus Wagner in Comparative Political Studies

## Data Availability

Replication materials and code can be found at Hübscher et al. (2023b).

## Notes

1. Our analysis, therefore, does not directly examine the effect of IMF intervention on political stability, though we also examine the effects of the IMF on support for incumbents to the extent that our approach allows us to do so.

2. To give an example, Bojar et al. (2022) find that voters punish governments more for austerity when the IMF intervenes. But it is plausible that the presence of the IMF in their analysis simply reflects that the crisis was more severe, and therefore, the austerity package was more encompassing compared to the countries and periods that experienced austerity without the IMF. Our analysis will disentangle these different aspects of IMF interventions.

3. This disappointment extends to the inability of the government to solve fundamental problems, such as corruption, that may have caused the crisis. The credibility and the competence logics are theoretically distinct, but both positively influence the expectations of voters that the crisis will be resolved. We, therefore, subsume them under the same class of arguments highlighting the impact of the IMF on economic stability.

4. During the Eurozone crisis, governments in crisis countries interacted with the so-called Troika, which consisted of representatives from the IMF, the European Central Bank, and the European Commission.

5. Fianna Fail and Fine Gael in Ireland, Pasok and Syriza in Greece, PS and PSD in Portugal, and PP and PSOE in Spain.

6. We also asked respondents if they would vote for the government after this announcement, which they could respond with “*Yes*” or “*No*.” In the analyses presented in the main text, we concentrate on policy approval but show the results for vote intention in Appendix E (see Figure E.1).

7. In our pre-analysis plan, we also formulated expectations about two other mechanisms, government accountability and competence. The corresponding questions were included in our survey, but they showed to be irrelevant. For the sake of transparency, we included the results in the appendix (Section F.3, Figure F.6) because we think that they do not contribute sufficiently to our empirical understanding of the underlying mechanism.

8. Replication materials and code can be found at Hübscher et al. (2023b).

9. We decided to constrain our analysis to Europe instead of selecting countries from other parts of the world where the IMF has intervened regularly, for example, Latin America. By focusing on Europe, we limit the contextual variation because the IMF interventions happened roughly at the same time (between 2010 and 2012) and the interventions were a result of the same, overarching economic and sovereign debt crisis in the Eurozone.

10. The original data provide information on the conditions listed in the memorandum of understanding (MoU) between the IMF and the respective country. For the purpose of this paper, some policy areas were merged and data recoded (see the Appendix B for details). Since the financial support for the Spanish financial sector was provided by the EU, there is no formal MoU between the IMF and Spain. We, therefore, coded the agreements between the EU and Spain ourselves using the same coding scheme as the one used by Kentikelenis et al. (2016).

11. Soft conditions can usually be changed retrospectively, while hard conditions represent the reforms that a country has to implement independent of potential changes in government. Because the data by Kentikelenis et al. (2016) end in 2014, the figures cover the period between 2010 and 2014.

12. In terms of the total number of measures a country had to implement, Greece stands out—also in comparison to the average number of measures imposed on OECD and non-OECD countries alike. This is partially explained by the fact that we show the numbers that combine the different IMF rescue packages in the case of Greece.

13. Also note that there is a large fraction of respondents who strongly disagree with fiscal adjustment, that is, who chose 0, while only few strongly agree, that is, who chose 10 on the 11-point scale. This is especially the case for Greece and Spain and to a lesser extent in Portugal and Ireland. In the latter three countries, the IMF treatment has a fairly strong effect on those who strictly oppose fiscal adjustment, but not in Greece.

14. Full regression tables are included in Appendix D.

15. The five statements are: *“Governments should redistribute income from the better off to those who are less well off”*; *“Major public services and industries ought to be in state ownership”*; *“Government should take more responsibility to ensure that everyone is provided for”*; *“Competition is bad and mostly favors the strongest”*; and *“People can only get rich at the expense of others.”* Opinions are measured on a standard 5-point agree–disagree scale. The scale was reversed so higher values are more right-wing to be in alignment with the general left-right scale.

16. Mainstream parties are PASOK, ND, To Potami, Enosi Kentroon, and Dimar (Greece); PS, PSD, and CDS-PP (Portugal); and PSOE, PP, and Ciudadanos (Spain). Radical parties are Syriza, ANEL, Golden Dawn, KKE, and Laiki Enotita (Greece); BE and PCP (Portugal); and Podemos and VOX (Spain). Due to the lack of a sufficient number of observations on “radical parties” in Ireland, the country was excluded from this particular analysis.

17. In the appendix, we present the results for HTE of ideology on the efficiency mechanism (Figure F.9). There is evidence that—on average—respondents who are more leftist expect a more efficient crisis solution with the IMF than without the IMF. The difference between more leftist and right-wing voters, however, is statistically not significant.

18. The results for the two other mechanisms (competence and accountability) that we tested are in the Appendix (section F.3). The IMF treatment does not affect the measures capturing “competence” or “democratic accountability.”

19. Consistent with our previous results, the two other perceptions that we tested are not relevant mediators: the effect of IMF involvement does not occur via either accountability or competence.
