# Supplementary material for: Voters and the IMF: Experimental Evidence From European Crisis Countries
Source: Comp Polit Stud. 2023 Oct 13;57(11):1870–901. doi: 10.1177/00104140231204229 (PMC11300164; doi:10.1177/00104140231204229)
Supplement: Supplemental Material - Voters and the IMF: Experimental Evidence From European Crisis Countries [file sj-pdf-1-cps-10.1177_00104140231204229.pdf]

Online Appendix for

“Voters and the IMF: Experimental Evidence From European Crisis Countries”

Forthcoming in *Comparative Political Studies*

Evelyne Hübscher
Central European University
huebschere@ceu.edu

Thomas Sattler
University of Geneva
thomas.sattler@unige.ch

Markus Wagner
University of Vienna
markus.wagner@univie.ac.at

1 Design of Survey Experiment

Respondents were presented with a fictional scenario that varied in four randomly varying attributes: crisis severity, proposed spending cuts, main party in government/opposition, and IMF involvement. The main text of the vignette was presented over a series of screens to make it easier to absorb the information. The text in square brackets ([]) varies in the treatment levels listed, except for the country. The IMF treatment consists of whether *Screen 4* is shown or not, and in whether respondents are reminded of the IMF involvement in *Screen 5*.

All treatments in *Screen 2* vary together and describe the public debt situation. In *Screen 3*, the two government/opposition treatments vary together, as do the two spending cuts treatments.

The text of the vignettes is as follows:

Screen 1: We are going to describe a situation [Greece/Ireland/Portugal/Spain] could face in the future, in 2026. Some parts of the description may seem important to you; other parts may seem unimportant.

Screen 2: Here is some background: In 2026, [Greece/Ireland/Portugal/Spain] is experiencing a [moderate/massive] increase in the level of public debt. The debt level is [somewhat/extremely] troubling. The government is finding it [fairly/very] difficult to borrow money on financial markets to pay for public spending. This presents a [somewhat/highly] urgent threat to the security of your savings and to the broader economic situation in [Greece/Ireland/Portugal/Spain].

Screen 3: Here is some more background: To address the economic concerns, the [Fianna Fáil/Fine Gael], [PASOK/New Democracy], [Partido Social Democrata/Partido Socialista], [Partido Popular/PSOE] Prime Minister announces in a televised speech that the government will implement [moderate/massive] spending cuts to limit the increase in public debt. These cuts will [slightly/deeply] affect funding for pensions, health care, education and public transport, for instance. The main opposition party, [Fine Gael/Fianna Fáil], [New Democracy/PASOK], [Partido Socialista/Partido Social Democrata], [PSOE/Partido Popular], criticizes the measures and doubts whether they will be successful.

Screen 4: Here is some additional background:

The Prime Minister says that these spending cuts are necessary. This is because the International Monetary Fund (IMF) has made these cuts a precondition for [Greece/Ireland/Portugal/Spain] to get an emergency loan that could stabilise the financial situation. The IMF is an international organization that provides emergency loans to countries in crisis, but only to governments who commit to carry out certain reforms. [Greece/Ireland/Portugal/Spain] would not receive the IMF loan without the cuts.]
Screen 5: Here is a summary of the situation again, for your reference:

- COUNTRY has experienced a [moderate/massive] increase in the level of public

debt.

- The Prime Minister is a member of [Fianna Fáil/Fine Gael], [PASOK/New Democracy], [Partido Social Democrata/Partido Socialista], [Partido Popular/PSOE] .
- The government will implement [moderate/massive] spending cuts to limit the increase in public debt.
- **ONLY SHOWN TO TREATMENT GROUP**: The Prime Minister says that these spending cuts represent the precondition for an emergency loan from the International Monetary Fund (IMF).

Outcome Questions

- General level of policy approval: *To what extent would you approve of the Prime Minister's announcement to impose spending cuts in response to the debt crisis?* (Response categories: 'Strongly disapprove' (0) to 'Strongly approve' (10))
- Vote intention: *Would you vote for the government after this announcement?* (Response categories: 'Yes', 'No')

Before answering the mediating questions, the respondents were again reminded about the content of the vignette (same as Screen 5).

Mediating Questions

- Competence: *Do you think that, in this debt crisis, the government proved to be a competent or an incompetent manager of the economy?* (Response categories: 'Completely incompetent' (0) to 'Completely competent' (10))
- Assessment of Policy Success: *Do you think the government's decision to cut spending will be successful or unsuccessful in resolving the debt crisis?* (Response categories: 'Completely unsuccessful' (0) to 'Completely successful' (10))
- Sovereignty: *To what extent do you think that the government was free to choose its own response to this debt crisis?* 8Response categories: 'Entirely unfree' (0) to 'entirely free' (10)).
- Accountability: *To what extent do you think that the decision to cut spending matches the views of the Irish [Spanish, Portuguese, Greek] voters?* (Response categories: 'Not at all' (0) to 'fully' (10)).

2 IMF Conditionality Data

The figures presented in section 3.2 are based on the data collected by Kentikelenis et al. (?). For Spain, we coded the 'Memorandum of Understanding' (from July 20, 2012) using the same coding instructions as suggested by in their code book on p. 15. All the policy changes mandated in Spain were linked to reforms of the banking system, the

financial infrastructure, and monetary policy issues.

For the ease of presentation, we cumulated some policy categories, specifically: the categories 'privatization' and 'state-owned enterprises' were put into one. The categories for 'fiscal issues' and revenue/taxation' were also combined. No policy reforms were categorized under the following dimensions: environment, external sector & trade, redistributive policies, and social policies.

3 Descriptives & Balance Test

These tables show that the treatment and control groups do not differ on key covariates (gender, age, ideology and education) and present the simple bivariate difference in the key outcome variable between treatment and control groups.

Table 1: Balance btw *'treated'* vs. *'non-treated'* group

	IMF treatment		
	no	yes	Total
Female			
Mean	0.50	0.48	0.49
Number of nonmissing values	2562.00	2655.00	5217.00
Age			
Mean	3.87	3.89	3.88
Standard deviation	1.32	1.33	1.33
Number of nonmissing values	2562.00	2655.00	5217.00
Left-Right Scale			
Mean	4.80	4.84	4.82
Standard deviation	2.33	2.33	2.33
Number of nonmissing values	2545.00	2649.00	5194.00
Education, 3 categories			
Mean	2.37	2.34	2.36
Standard deviation	0.67	0.68	0.68
Number of nonmissing values	2537.00	2624.00	5161.00
Income, 4 categories			
Mean	2.18	2.16	2.17
Standard deviation	1.11	1.11	1.11
Number of nonmissing values	2442.00	2522.00	4964.00

Table 2: Descriptives of 'Approval'-Variable (key DV)

	Full	Greece	Portugal	Spain	Ireland
Difference	0.27	-0.11	0.22	0.38	0.61
SE	0.07	0.16	0.14	0.14	0.15
Control	4.23	4.03	4.47	3.93	4.53
N(Control)	2560	644	628	687	601
Treatment	4.50	3.93	4.69	4.31	5.14
N(Treatment)	2654	676	659	696	623
N(Overall)	5212	1318	1285	1381	1222

4 Regression Tables

The figures included in the main text of the paper are based on the following regression tables. Specifically, Figure **??** draws on table 3, and Figure **??** draws on table 4. The ACME visualized in Figure **??** are based on table 5.

Table 3: Regression Results: Approval of Policy

	Overall	Greece	Portugal	Spain	Ireland
IMF treatment	0.288***	-0.0451	0.236	0.405**	0.572***
	(0.0634)	(0.130)	(0.126)	(0.128)	(0.122)
Public debt	-0.0283	0.149	-0.294*	0.0133	0.00520
	(0.0634)	(0.129)	(0.126)	(0.128)	(0.122)
Party in Gov	0.0689	-0.155	0.152	0.234	0.0253
	(0.0815)	(0.168)	(0.170)	(0.161)	(0.153)
Spending cuts	-0.278***	-0.174	-0.296*	-0.295*	-0.328**
	(0.0634)	(0.130)	(0.126)	(0.129)	(0.123)
Female	-0.222***	-0.202	-0.278*	-0.0750	-0.293*
	(0.0644)	(0.130)	(0.130)	(0.131)	(0.126)
Age (7 cats)	-0.137***	-0.0890	-0.133**	-0.219***	-0.103*
	(0.0245)	(0.0524)	(0.0491)	(0.0497)	(0.0464)
L-R self-placement	0.197***	0.125***	0.177***	0.235***	0.177***
	(0.0147)	(0.0311)	(0.0321)	(0.0311)	(0.0335)
Public sector	-0.196*	-0.202	-0.358*	-0.128	-0.0388
	(0.0772)	(0.163)	(0.152)	(0.162)	(0.143)
Income, 2nd quartile	0.0564	-0.171	0.190	0.216	-0.0866
	(0.0867)	(0.172)	(0.171)	(0.182)	(0.170)
Income, 3rd quartile	0.0738	-0.0646	0.143	0.193	-0.0349
	(0.0903)	(0.180)	(0.203)	(0.184)	(0.166)
Income, 4th quartile	0.273**	0.305	0.363	0.333	0.0897
	(0.102)	(0.233)	(0.190)	(0.185)	(0.247)
Income, refused	-0.222	0.131	-0.414		-0.306
	(0.157)	(0.283)	(0.320)		(0.229)
Gov voter	0.780***	1.150**	0.357	1.100***	1.016***
	(0.125)	(0.379)	(0.218)	(0.264)	(0.223)
Opp voter	0.130	0.434*	0.152	-0.126	0.189
	(0.115)	(0.220)	(0.238)	(0.234)	(0.258)
ISCED 3-5	-0.279*	-0.609	-0.0454	-0.543**	0.0164
	(0.111)	(0.406)	(0.225)	(0.187)	(0.203)
ISCED 6-8	-0.143	-0.535	0.164	-0.456*	0.141
	(0.113)	(0.406)	(0.227)	(0.185)	(0.223)
ISCED missing	-0.417	-1.094	-0.170	-0.452	-0.189
	(0.329)	(0.914)	(0.880)	(0.632)	(0.484)
Economic ideology	0.328***	0.494***	0.176*	0.262**	0.309***
	(0.0426)	(0.0870)	(0.0893)	(0.0834)	(0.0853)
Trust, national	0.271***	0.284***	0.248***	0.253***	0.302***
	(0.0191)	(0.0433)	(0.0384)	(0.0377)	(0.0385)
Trust, international	0.203***	0.269***	0.135***	0.179***	0.190***
	(0.0191)	(0.0394)	(0.0404)	(0.0376)	(0.0379)
Gov party x Gov vote	-0.120	-0.186	0.146	-0.407	-0.501
	(0.168)	(0.430)	(0.346)	(0.362)	(0.346)
Gov party x Opp vote	-0.402*	-0.0466	-0.773*	-0.833*	0.114
	(0.166)	(0.427)	(0.320)	(0.368)	(0.329)
Portugal	0.177				
	(0.0960)				
Spain	0.357***				
	(0.0948)				
Ireland	0.561***				
	(0.0968)				
Constant	1.175***	1.022	2.379***	1.845***	1.373**
	(0.240)	(0.593)	(0.488)	(0.455)	(0.452)
N	5190	1319	1287	1368	1216

Standard errors in parentheses. * $p < 0.05$, ** $p < 0.01$, *** $p < 0.001$

Table 4: Regression Results: Heterogeneous Treatment Effects

	LR/Trust	Econ ideology	Gov match	Radical vote
IMF Treatment	0.365*	0.630*	0.308***	0.380
	(0.179)	(0.252)	(0.0814)	(0.219)
L-R self-placement	0.222***		0.197***	0.197***

– continued from previous page.

	LR/Trust	Econ ideology	Gov match	Radical vote
	(0.0204)		(0.0147)	(0.0183)
Economic ideology	0.327***	0.552***	0.327***	0.328***
	(0.0426)	(0.0589)	(0.0426)	(0.0561)
Trust, international	0.186***	0.219***	0.203***	0.216***
	(0.0237)	(0.0238)	(0.0191)	(0.0254)
Treatment x LR position	-0.0475			
	(0.0276)			
Treatment x Econ ideology		-0.173*		
		(0.0814)		
Treatment x Trust, int	0.0333	0.0277		
	(0.0273)	(0.0274)		
Gov voter	0.779***	0.897***	0.847***	0.514
	(0.125)	(0.127)	(0.151)	(0.290)
Opp voter	0.131	0.291*	0.118	-0.126
	(0.115)	(0.116)	(0.141)	(0.279)
Treatment x Gov vote			-0.130	-0.0180
			(0.165)	(0.383)
Treatment x Opp vote			0.0264	0.0664
			(0.163)	(0.382)
Mainstream voter				0.258
				(0.283)
Radical voter				0.0212
				(0.197)
Treatment x Mainstream				-0.211
				(0.407)
Treatment x Radical				-0.305
				(0.271)
Public debt	-0.0278	-0.0299	-0.0270	-0.107
	(0.0634)	(0.0643)	(0.0634)	(0.0843)
Spending cuts	-0.279***	-0.278***	-0.278***	-0.330***
	(0.0634)	(0.0643)	(0.0634)	(0.0842)
Female	-0.223***	-0.277***	-0.224***	-0.186*
	(0.0644)	(0.0652)	(0.0644)	(0.0860)
Age (7 cats)	-0.137***	-0.140***	-0.137***	-0.137***
	(0.0245)	(0.0248)	(0.0245)	(0.0325)
Public sector	-0.195*	-0.211**	-0.196*	-0.245*
	(0.0772)	(0.0783)	(0.0772)	(0.103)
Income, 2nd quartile	0.0558	0.0284	0.0571	0.135
	(0.0867)	(0.0880)	(0.0868)	(0.115)
Income, 3rd quartile	0.0752	0.101	0.0731	0.145
	(0.0903)	(0.0916)	(0.0903)	(0.122)
Income, 4th quartile	0.268**	0.303**	0.273**	0.351**
	(0.102)	(0.104)	(0.102)	(0.128)
Income, refused	-0.224	-0.223	-0.221	-0.118
	(0.156)	(0.159)	(0.157)	(0.277)
ISCED 3-5	-0.274*	-0.344**	-0.278*	-0.374*
	(0.111)	(0.112)	(0.111)	(0.151)
ISCED 6-8	-0.137	-0.251*	-0.142	-0.304*
	(0.113)	(0.114)	(0.113)	(0.151)
ISCED missing	-0.429	-0.533	-0.422	-0.437
	(0.329)	(0.335)	(0.329)	(0.521)
Trust, national	0.270***	0.259***	0.270***	0.251***
	(0.0191)	(0.0194)	(0.0191)	(0.0257)
Gov party x Gov vote	-0.119	-0.0804	-0.122	0.0538
	(0.168)	(0.171)	(0.168)	(0.211)
Gov party x Opp vote	-0.405*	-0.494**	-0.403*	-0.516*
	(0.166)	(0.169)	(0.166)	(0.211)
Portugal	0.179	0.0319	0.179	0.105
	(0.0960)	(0.0970)	(0.0960)	(0.117)
Spain	0.363***	0.152	0.358***	0.167
	(0.0949)	(0.0950)	(0.0949)	(0.115)
Ireland	0.566***	0.499***	0.562***	
	(0.0968)	(0.0982)	(0.0968)	
Constant	1.132***	1.715***	1.164***	1.394***
	(0.253)	(0.267)	(0.241)	(0.352)

– continued from previous page.

	LR/Trust	Econ ideology	Gov match	Radical vote
N	5190	5213	5190	3139

Standard errors in parentheses. * $p < 0.05$, ** $p < 0.01$, *** $p < 0.001$

Table 5: ACME – All mediators

	Mediated effect	95 CI:Lower	95 CI:Upper	Direct effect	95 CI:Lower	95 CI:Upper
**Effectiveness**	0.12	0.04	0.19	0.17	0.07	0.27
Effectiveness(Greece)	0.03	-0.14	0.18	-0.08	-0.27	0.12
Effectiveness(Portugal)	0.07	-0.07	0.21	0.15	-0.04	0.35
Effectiveness(Spain)	0.17	0.03	0.32	0.24	0.04	0.43
Effectiveness(Ireland)	0.18	0.05	0.31	0.39	0.20	0.59
**Sovereignty**	-0.04	-0.06	-0.03	0.33	0.20	0.45
Sovereignty(Greece)	-0.05	-0.09	-0.02	-0.00	-0.25	0.24
Sovereignty(Portugal)	-0.05	-0.11	-0.00	0.28	0.04	0.52
Sovereignty(Spain)	-0.01	-0.03	0.00	0.42	0.17	0.67
Sovereignty(Ireland)	-0.04	-0.09	-0.01	0.62	0.39	0.86
**Competence**	0.02	-0.05	0.09	0.26	0.16	0.37
Competence(Greece)	-0.09	-0.26	0.07	0.03	-0.15	0.22
Competence(Portugal)	-0.06	-0.21	0.07	0.29	0.09	0.49
Competence(Spain)	0.03	-0.09	0.15	0.38	0.16	0.59
Competence(Ireland)	0.18	0.04	0.32	0.40	0.21	0.59
**Accountability**	0.00	-0.05	0.05	0.29	0.17	0.40
Accountability(Greece)	0.01	-0.10	0.11	-0.06	-0.29	0.16
Accountability(Portugal)	-0.03	-0.12	0.06	0.26	0.03	0.48
Accountability(Spain)	-0.02	-0.13	0.08	0.43	0.21	0.66
Accountability(Ireland)	0.08	-0.04	0.19	0.50	0.29	0.70

5 Vote choice

The effect on vote choice is more complicated to present as we need to examine treatments conditional on whether each respondent is generally a supporter of the government or not. Recall that the government party varied randomly between the two largest parties in the system. Figure 1 shows the effect of IMF conditionality on whether respondents say they would vote for the government in the vignette. We can see that those who are generally supporters of the government party do not respond to the treatment. However, opposition supporters are more likely to vote for the government if the IMF demanded the policy programme. The treatment is statistically significant across the four countries and for Ireland, with substantively similar effects in Portugal and Spain. Smaller sample sizes mean statistical significance is harder to reach here. Interestingly, independent supporters (i.e. those who before the experiment do not voice support for either the government or the opposition) do not respond to the treatment.

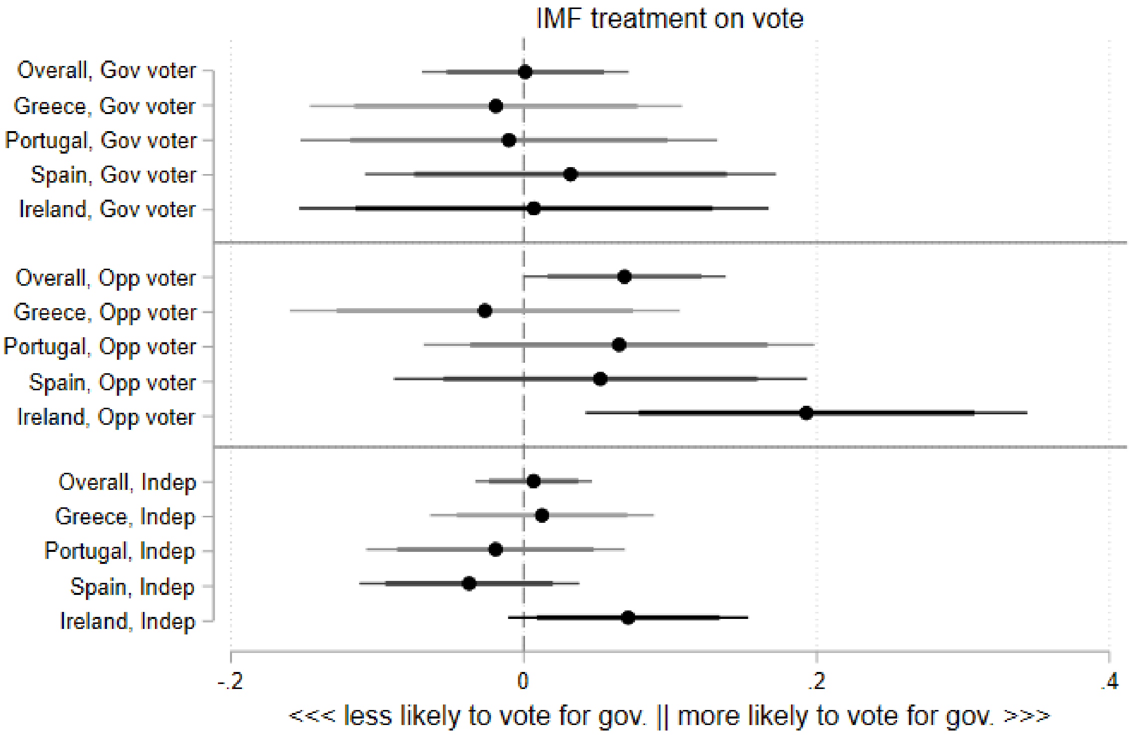

Figure 1: Effect of IMF conditionality on vote choice

6 Additional Analyses

6.1 Effects of other treatments

Figure 2 shows that the other treatments have varying effects on policy approval. Recall that the effect of our main treatment of interest was about 0.3 overall. The size of the public debt – in other words, the severity of the crisis – has no impact on policy approval, though Portuguese respondents react somewhat negatively to this treatment. Spending cuts lead to lower policy approval, though this effect is not quite statistically significant at the 0.05 level. Interestingly, Portuguese respondents respond positively to cuts. Finally, the effects of which party is said to be in government depend on which party each respondent supports. Specifically, those who support the opposition are less likely to support the policy.

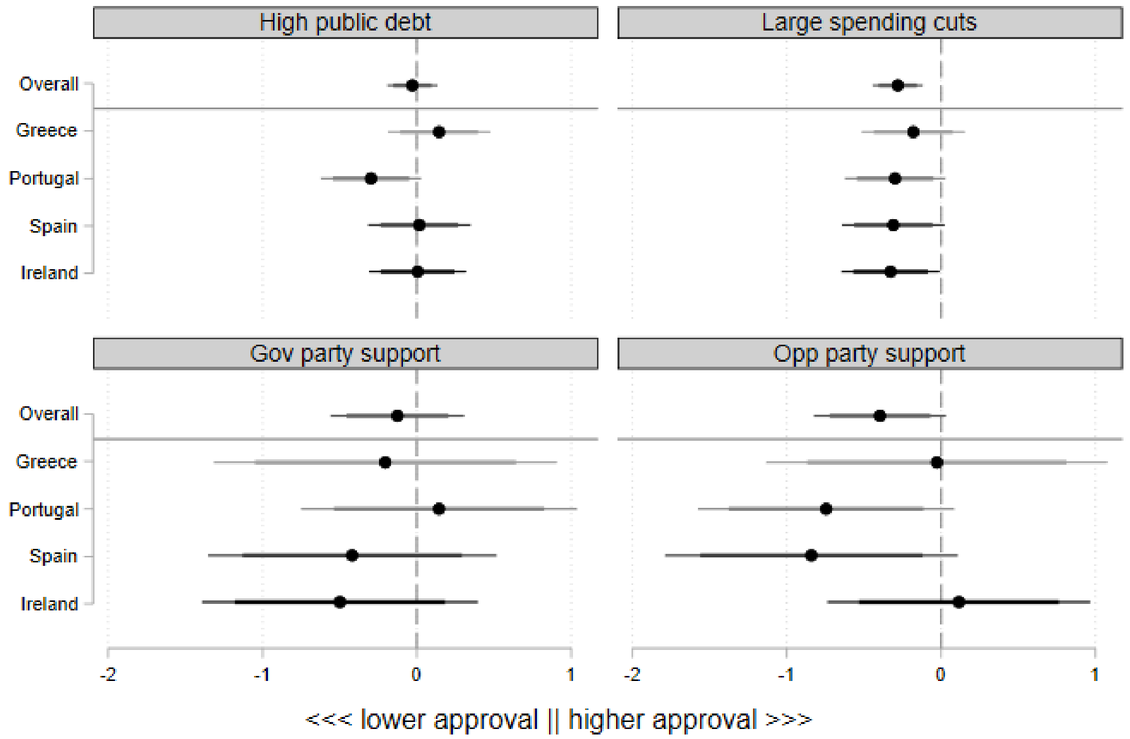

Figure 2: Effect of other treatments on policy approval

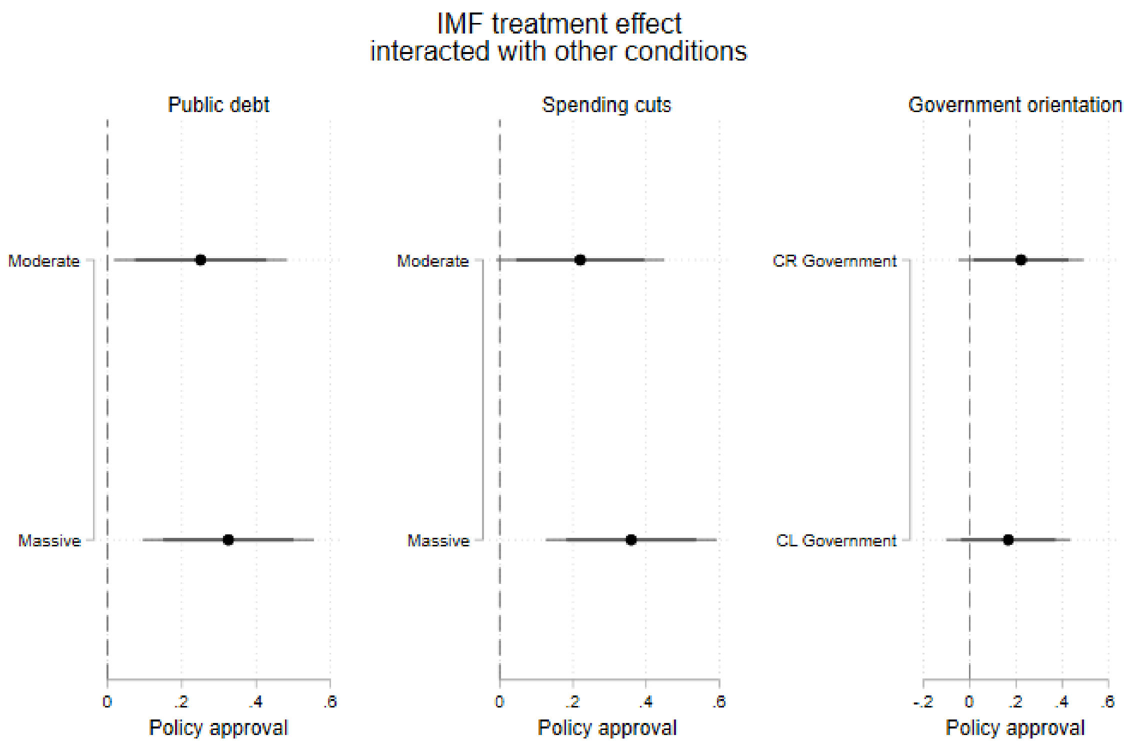

Figure 3: IMF Treatment interacted with contextual conditions (economic and political)

Table 6: Probability Success

	Overall	Greece	Portugal	Spain	Ireland
IMF treatment	0.181**	0.0426	0.112	0.271*	0.311**
	(0.0591)	(0.122)	(0.109)	(0.123)	(0.119)
Public debt	-0.0822	-0.138	-0.185	-0.0112	0.00843
	(0.0591)	(0.121)	(0.108)	(0.123)	(0.119)
Party in Gov	0.0188	-0.272	0.365*	-0.0301	0.0387
	(0.0760)	(0.158)	(0.146)	(0.155)	(0.149)
Spending cuts	-0.138*	-0.00867	-0.191	-0.199	-0.166
	(0.0591)	(0.122)	(0.109)	(0.124)	(0.120)
Female	-0.0631	-0.117	-0.0433	-0.0618	-0.0399
	(0.0601)	(0.123)	(0.112)	(0.126)	(0.124)
3.Q2_3	-0.235*	-0.420*	-0.364*	-0.125	-0.0312
	(0.0939)	(0.194)	(0.174)	(0.205)	(0.181)
4.Q2_3	-0.311***	-0.288	-0.484**	-0.315	-0.108
	(0.0901)	(0.188)	(0.167)	(0.193)	(0.177)
5.Q2_3	-0.415***	-0.446*	-0.673***	-0.292	-0.298
	(0.0926)	(0.192)	(0.165)	(0.201)	(0.188)
6.Q2_3	-0.446***	-0.351	-0.564**	-0.464*	-0.467*
	(0.106)	(0.234)	(0.199)	(0.216)	(0.210)
7.Q2_3	-0.221			-0.378	
	(1.509)			(1.622)	
L-R self-placement	0.114***	0.117***	0.0931***	0.0987***	0.114***
	(0.0137)	(0.0296)	(0.0277)	(0.0299)	(0.0328)
Public sector	-0.0819	-0.0866	-0.243	0.0798	-0.0130
	(0.0720)	(0.155)	(0.131)	(0.156)	(0.140)
Income, 2nd quartile	0.0373	-0.0934	0.202	0.311	-0.313
	(0.0809)	(0.162)	(0.147)	(0.175)	(0.166)
Income, 3rd quartile	0.0443	-0.0363	0.263	0.112	-0.117
	(0.0842)	(0.170)	(0.174)	(0.177)	(0.163)
Income, 4th quartile	0.160	0.312	0.263	0.227	-0.324
	(0.0955)	(0.220)	(0.164)	(0.178)	(0.242)
Income, refused	-0.197	0.0687	-0.347		-0.388
	(0.146)	(0.267)	(0.275)		(0.224)
Gov voter	0.937***	1.210***	0.678***	1.346***	1.016***
	(0.117)	(0.356)	(0.188)	(0.254)	(0.219)
Opp voter	0.114	0.176	0.190	-0.0927	0.269
	(0.107)	(0.207)	(0.205)	(0.224)	(0.253)
ISCED 3-5	-0.205*	-0.489	-0.0981	-0.238	-0.226
	(0.103)	(0.385)	(0.193)	(0.179)	(0.199)
ISCED 6-8	-0.192	-0.499	-0.0782	-0.209	-0.287
	(0.105)	(0.384)	(0.195)	(0.179)	(0.219)
ISCED missing	-0.293	-1.018	-0.355	-0.171	-0.129
	(0.307)	(0.860)	(0.757)	(0.606)	(0.474)
Economic ideology	0.198***	0.249**	0.0884	0.225**	0.205*
	(0.0398)	(0.0819)	(0.0768)	(0.0803)	(0.0835)
Trust, national	0.315***	0.292***	0.324***	0.298***	0.327***
	(0.0178)	(0.0408)	(0.0331)	(0.0362)	(0.0377)
Trust, international	0.181***	0.223***	0.153***	0.159***	0.193***
	(0.0178)	(0.0371)	(0.0348)	(0.0361)	(0.0372)
Gov party x Gov vote	-0.126	0.00202	-0.0476	-0.661	-0.551
	(0.157)	(0.405)	(0.298)	(0.347)	(0.338)
Gov party x Opp vote	-0.308*	0.461	-0.536	-0.957**	-0.116
	(0.155)	(0.403)	(0.276)	(0.353)	(0.322)
Portugal	0.321***				
	(0.0895)				
Spain	0.346***				
	(0.0885)				
Ireland	0.453***				
	(0.0902)				
_cons	1.179***	1.444**	1.995***	1.548***	1.413***
	(0.212)	(0.531)	(0.396)	(0.408)	(0.419)
N	5190	1319	1287	1368	1216

Standard errors in parentheses. * $p < 0.05$, ** $p < 0.01$, *** $p < 0.001$

Table 7: Accountability Results

	Overall	Greece	Portugal	Spain	Ireland
IMF treatment	0.00347	0.0343	-0.0571	-0.0490	0.153
	(0.0607)	(0.127)	(0.123)	(0.118)	(0.119)
Public debt	-0.0801	-0.0446	-0.154	-0.171	0.0468
	(0.0607)	(0.126)	(0.123)	(0.118)	(0.119)
Party in Gov	-0.0536	-0.285	-0.0928	-0.0470	0.250
	(0.0780)	(0.164)	(0.166)	(0.148)	(0.150)
Spending cuts	-0.189**	-0.0581	-0.192	-0.202	-0.313**
	(0.0607)	(0.127)	(0.123)	(0.118)	(0.120)
Female	-0.208***	-0.268*	-0.0180	-0.269*	-0.279*
	(0.0617)	(0.128)	(0.127)	(0.120)	(0.124)
3.Q2_3	-0.0719	0.137	0.0672	-0.105	-0.311
	(0.0964)	(0.201)	(0.197)	(0.196)	(0.181)
4.Q2_3	-0.247**	0.0550	-0.0994	-0.269	-0.591***
	(0.0926)	(0.196)	(0.189)	(0.185)	(0.177)
5.Q2_3	-0.270**	-0.00556	-0.0843	-0.235	-0.754***
	(0.0950)	(0.200)	(0.188)	(0.192)	(0.188)
6.Q2_3	-0.231*	-0.213	0.275	-0.0761	-0.955***
	(0.109)	(0.244)	(0.225)	(0.206)	(0.210)
7.Q2_3	0.186			0.0590	
	(1.549)			(1.547)	
L-R self-placement	0.137***	0.137***	0.149***	0.0864**	0.185***
	(0.0141)	(0.0308)	(0.0314)	(0.0285)	(0.0328)
Public sector	0.0561	-0.0756	-0.156	0.153	0.230
	(0.0739)	(0.161)	(0.149)	(0.149)	(0.140)
Income, 2nd quartile	-0.106	-0.234	0.109	-0.0958	-0.186
	(0.0831)	(0.169)	(0.167)	(0.167)	(0.167)
Income, 3rd quartile	-0.0576	-0.138	0.258	-0.0900	-0.235
	(0.0864)	(0.177)	(0.197)	(0.169)	(0.163)
Income, 4th quartile	0.0909	0.229	0.436*	-0.0146	-0.620*
	(0.0981)	(0.229)	(0.186)	(0.170)	(0.242)
Income, refused	-0.245	0.0461	-0.246		-0.430
	(0.150)	(0.278)	(0.312)		(0.225)
Gov voter	0.582***	0.940*	0.465*	0.752**	0.769***
	(0.120)	(0.371)	(0.213)	(0.242)	(0.219)
Opp voter	-0.0440	-0.230	-0.0112	-0.0906	0.211
	(0.110)	(0.216)	(0.233)	(0.214)	(0.253)
ISCED 3-5	-0.347**	-0.440	-0.421	-0.335	-0.276
	(0.106)	(0.401)	(0.219)	(0.171)	(0.199)
ISCED 6-8	-0.416***	-0.571	-0.528*	-0.434*	-0.236
	(0.108)	(0.400)	(0.222)	(0.171)	(0.219)
ISCED missing	-0.240	-1.269	-0.939	-0.197	0.352
	(0.315)	(0.895)	(0.859)	(0.578)	(0.475)
Economic ideology	0.000949	-0.0400	-0.0552	0.115	0.00132
	(0.0408)	(0.0852)	(0.0872)	(0.0767)	(0.0836)
Trust, national	0.342***	0.320***	0.302***	0.353***	0.337***
	(0.0183)	(0.0424)	(0.0375)	(0.0346)	(0.0378)
Trust, international	0.127***	0.177***	0.130**	0.0806*	0.150***
	(0.0183)	(0.0386)	(0.0395)	(0.0344)	(0.0372)
Gov party x Gov vote	-0.343*	-0.502	-0.433	-0.651*	-0.469
	(0.161)	(0.422)	(0.338)	(0.331)	(0.339)
Gov party x Opp vote	-0.103	0.944*	-0.131	-0.468	-0.315
	(0.159)	(0.419)	(0.314)	(0.336)	(0.323)
Portugal	0.521***				
	(0.0919)				
Spain	0.937***				
	(0.0908)				
Ireland	0.324***				
	(0.0927)				
_cons	1.385***	1.449**	1.819***	2.586***	1.522***
	(0.217)	(0.552)	(0.450)	(0.389)	(0.420)
N	5190	1319	1287	1368	1216

Standard errors in parentheses. * $p < 0.05$, ** $p < 0.01$, *** $p < 0.001$

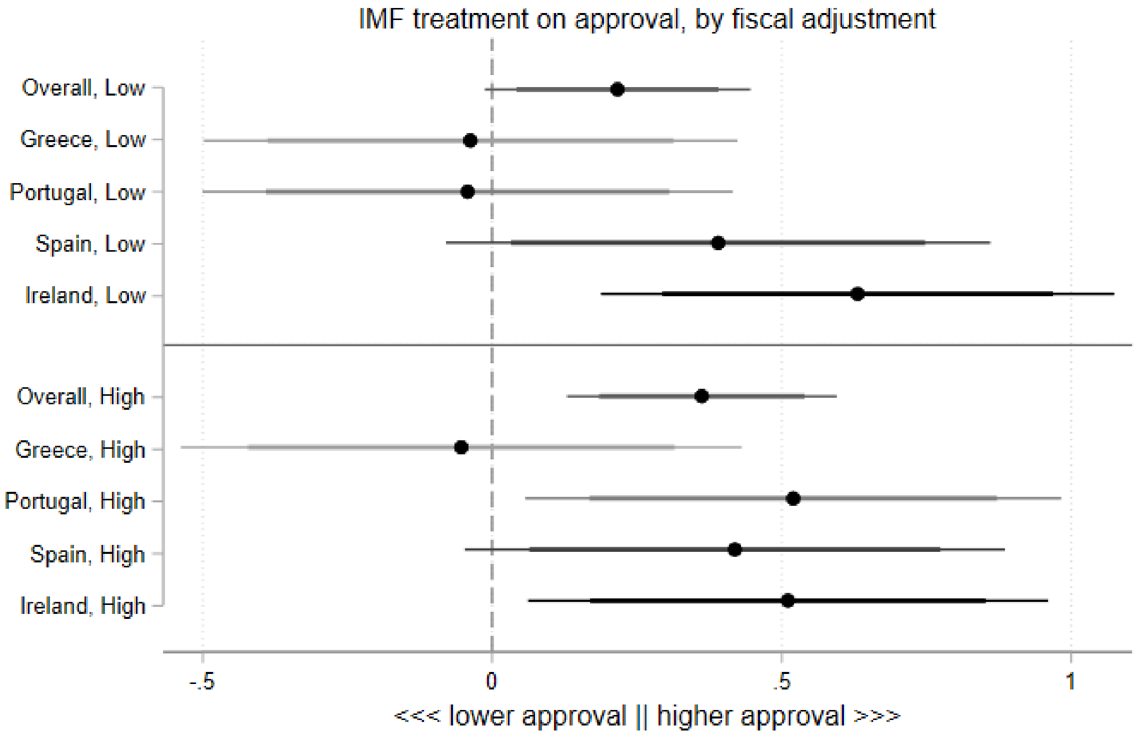

Figure 4: Effect of IMF conditionality on approval, conditioned on size of adjustment

6.2 Additional Heterogeneous Treatment Effects

The following graphs shows the heterogeneous treatment effect by voters' perceived government competence in addressing economic issues. The index is based on the answers to the following questions: Please indicate how confident you are that the government is able to deliver the following policy outcomes in the near future: a) protect jobs, b) preserve economic wealth, c) foster economic prosperity, d) balance the public budget. Respondents indicated their level of confidence on a scale from 1 (not at all confident) to 5 (very confident). The treatment effect is not clearly moderated by the perceived competence of the government in delivering positive economic outcomes.

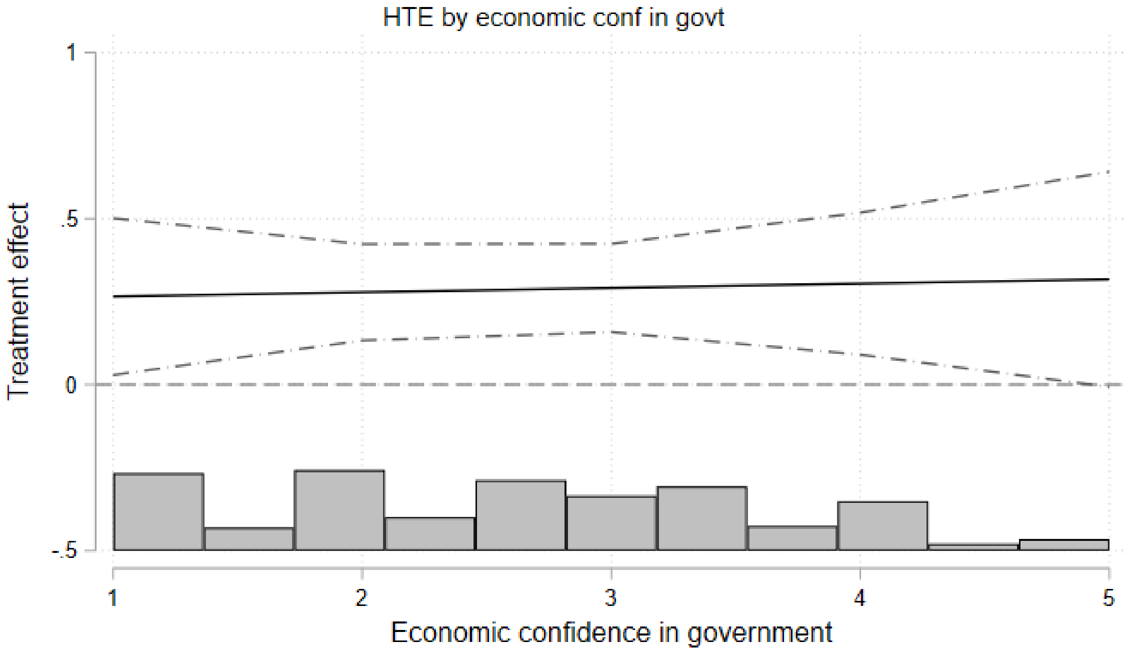

Figure 5: HTE: Perceived economic competence

6.3 Effect of other mediators on policy approval

As mentioned in section 3.1 (footnote no 6) we specified two additional mechanisms related to a) respondents' perception of government accountability, and b) respondents' perception of whether they expect the chosen austerity path to be successful. Figure 6 shows that the four mediators all have an impact on the outcome variable (approval). Respondents view policy packages more positively if they also believe that their government is competent and sovereign and that the policy package will be effective and represents voters well.

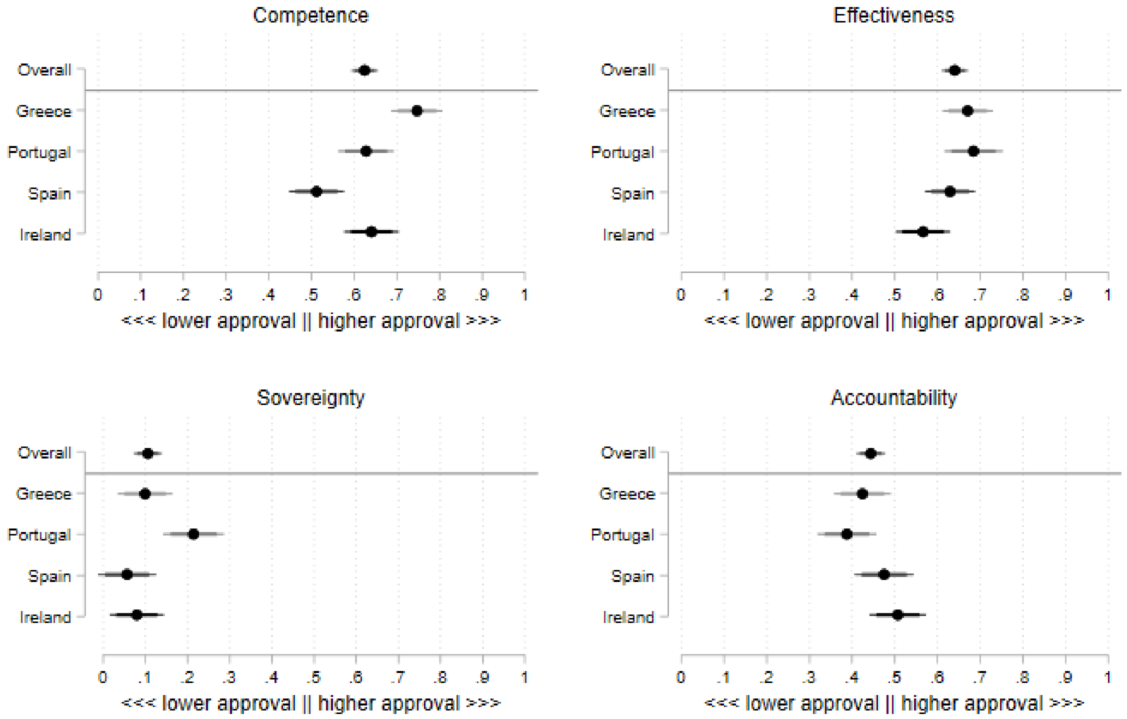

Figure 6: Effect of mediators on policy approval

While all mediators have an effect on policy approval, the relevant mechanisms for two of them, competence and accountability, proved to be irrelevant. Figures 7 and 8 show the corresponding results. Possibly, the reference to a scenario in the future influences perceptions of government competence,

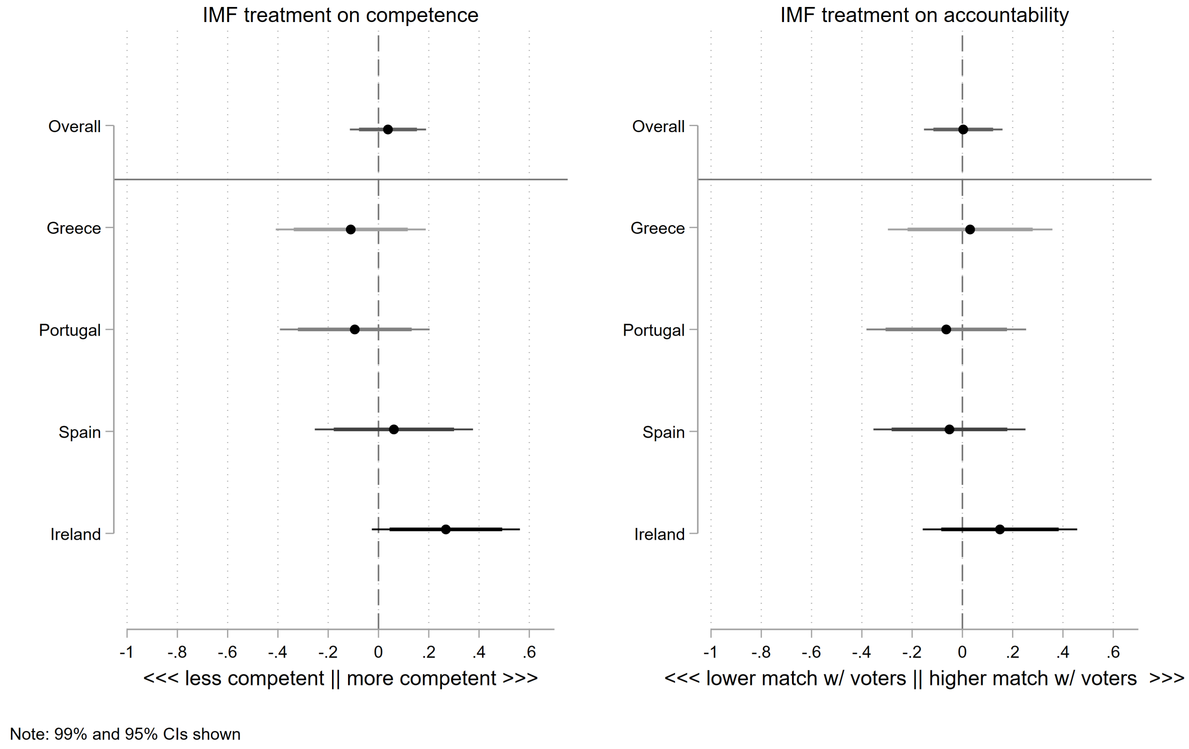

Figure 7: Treatment effects on additional mediators, by country

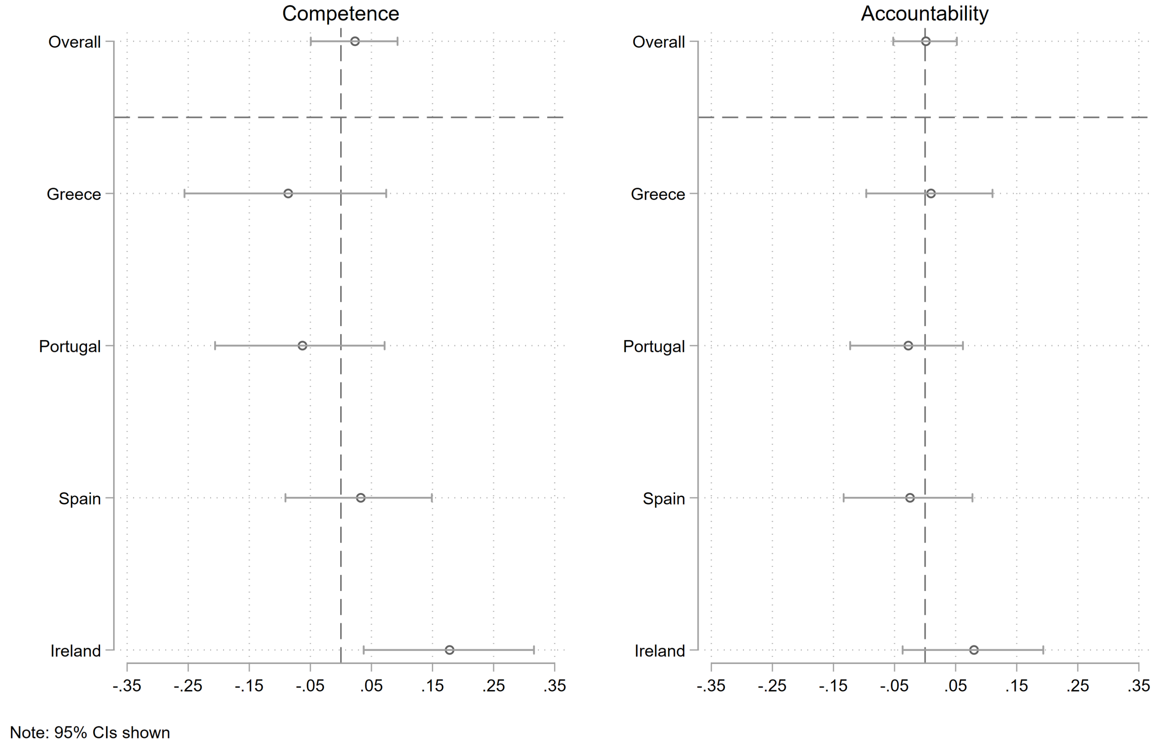

Figure 8: ACME for additional mechanisms, by country

Finally, Figure 9 and Figure 10 present the treatment effect of IMF intervention on the perceived effectiveness of the policy and the perceived sovereignty of the government, conditional on respondents' economic ideology. We can see that those with left-wing economic ideologies are more likely to think that IMF intervention will increase the effectiveness of a policy (though note that this interaction effect is not statistically significant), while the treatment effect on sovereignty is not (linearly) moderated by economic ideology.

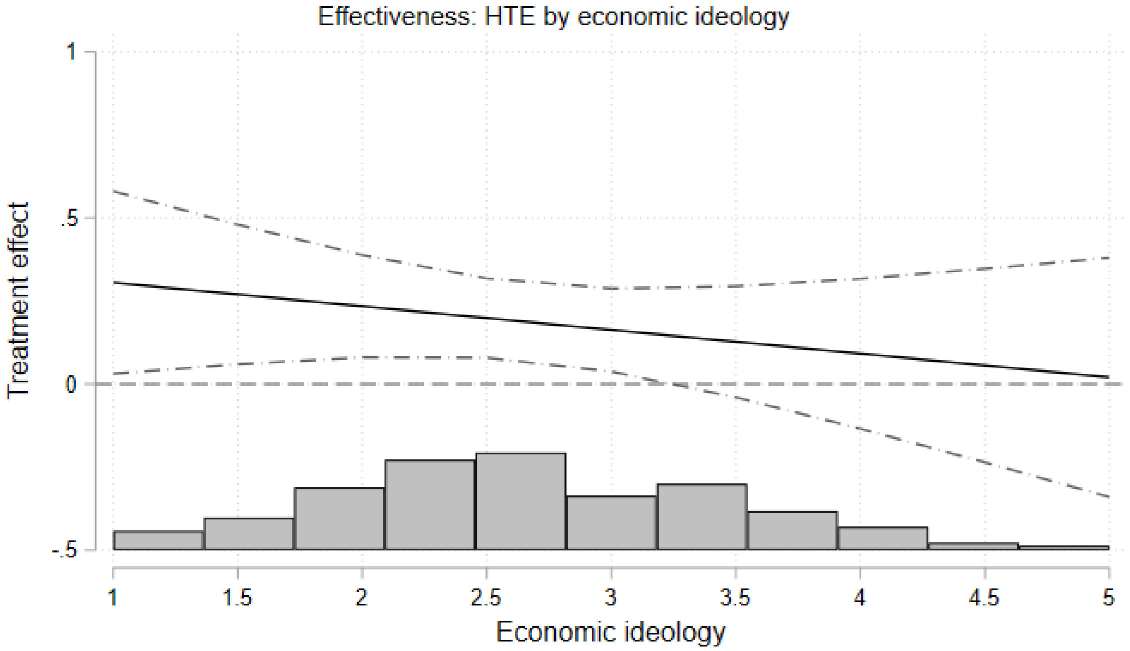

Figure 9: HTE: Efficiency by economic ideology

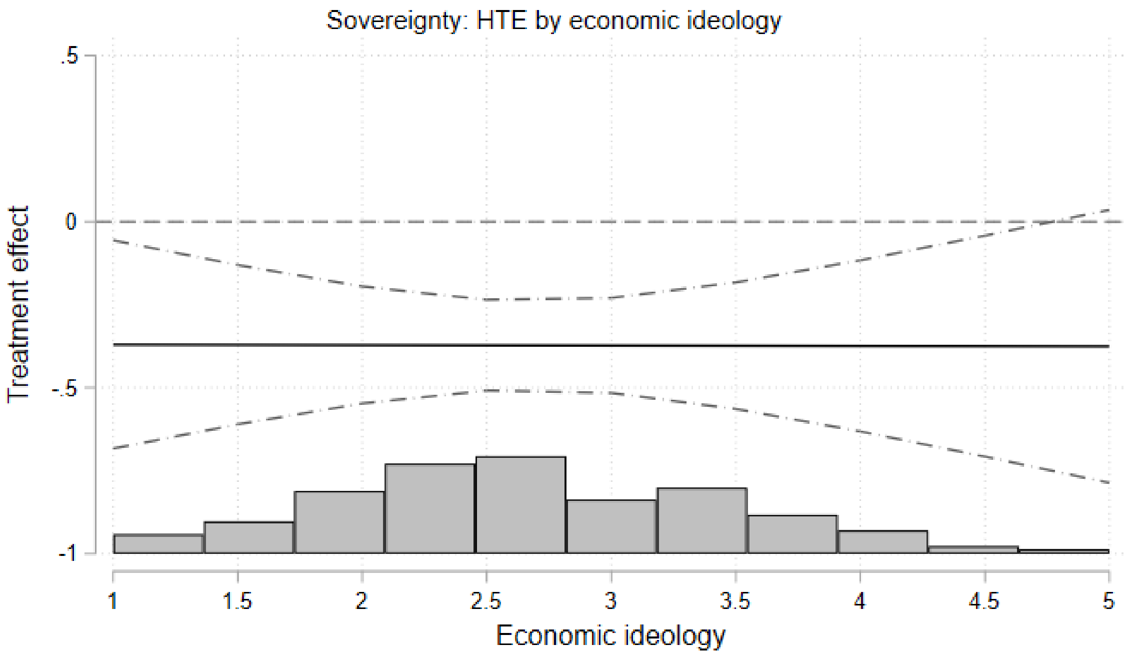

Figure 10: HTE: Sovereignty by Econ Ideology
